# Hypolithic communities shape soils and organic matter reservoirs in the ice-free landscapes of East Antarctica

**DOI:** 10.1038/s41598-020-67248-3

**Published:** 2020-06-24

**Authors:** Nikita Mergelov, Andrey Dolgikh, Ilya Shorkunov, Elya Zazovskaya, Vera Soina, Andrey Yakushev, Dmitry Fedorov-Davydov, Sergey Pryakhin, Alexander Dobryansky

**Affiliations:** 1 Institute of Geography, Russian Academy of Sciences, 119017 Moscow, Russia; 2 Faculty of Soil Science, Moscow State University, 119991 Moscow, Russia; 3 Institute of Physicochemical and Biological Problems in Soil Science, Russian Academy of Sciences, 142290 Pushchino, Russia; 4 Arctic and Antarctic Research Institute, 199397 Saint Petersburg, Russia

**Keywords:** Biogeochemistry, Ecology, Environmental sciences

## Abstract

The soils of East Antarctica have no rhizosphere with the bulk of organo-mineral interactions confined to the thin microbial and cryptogamic crusts that occur in open or cryptic niches and are collectively known as biological soil crust (BSC). Here we demonstrate that cryptic hypolithic varieties of BSC in the Larsemann Hills of East Antarctica contribute to the buildup of soil organic matter and produce several types of continuous organogenous horizons within the topsoil with documented clusters of at least 100 m^2^. Such hypolithic horizons accumulate 0.06–4.69% of organic carbon (TOC) with isotopic signatures (δ^13^C_org_) within the range of −30.2 – −24.0‰, and contain from 0 to 0.38% total nitrogen (TN). The properties of hypolithic organic matter alternate between cyanobacteria- and moss-dominated horizons, which are linked to the meso- and microtopography patterns and moisture gradients. The major part of TOC that is stored in hypolithic horizons has modern or centenary ^14^C age, while the minor part is stabilized on a millennial timescale through shallow burial and association with minerals. Our findings suggest that hypolithic communities create a “gateway” for organic carbon to enter depauperate soils of the Larsemann Hills and contribute to the carbon reservoir of the topsoil at a landscape level.

## Introduction

The ice-free landscapes of East Antarctica provide a very limited number of pathways for soil formation with only small amounts of organic matter accumulated in pedogenic environments^1–9^. Being partially isolated, oligotrophic, water and temperature restricted, exposed to high summer UV radiation and strong katabatic winds the terrestrial ecosystems of East Antarctica^10–12^ sustain no plants with root systems, which are known among the most powerful agents of pedogenesis. In the absence of rhizosphere and dramatically reduced downward migration of solutions, the vital organo-mineral interactions in soils are thought to be spatially confined to the thin areas of biofilms and crusts of microbial/cryptogamic origin. The latter are best known as biological soil crusts (BSC)^13,14^, and are also regarded as a part of cryptogamic ground covers of the planet^15^.

According to a global estimate^15^ the cryptogamic covers located both on terrestrial mineral surfaces and plants are responsible for nearly 7% of the net primary production by terrestrial vegetation and half of the biological nitrogen fixation on land. Their contribution to carbon and nitrogen cycles and specifically to the buildup of soil organic matter could differ significantly between biomes and still needs to be assessed at various scales.

Microbial and cryptogamic crusts are often distinguished as a separate component of the terrestrial ecosystem and more rarely considered as an integral part of the underlying soil being its organogenous horizon. However, as reviewed by J. Belnap^16^, a portion of both carbon and nitrogen fixed by BSC leaks to the surrounding soils and could increase its organic matter content. In polar and alpine environments, where decomposition rates are low, the BSC could continuously enrich soil in organic carbon^17^. In fact, BSC plays a number of roles typical of the common topsoil horizons like providing carbon and nitrogen “gateway” to soil, producing organic weathering agents, building physical structure of soil mainly through binding by extracellular polymeric substances (EPS), stabilizing soil and protecting it from erosion, raising soil fertility and stimulating plant growth^13,14,16^.

The primary production in soils of East Antarctica occurs mainly due to the free-living and lichenized cyanobacteria and green algae, as well as bryophytes which constitute BSC along with its heterotrophic components and are present both in open and cryptic niches^18,19^. Depending on the local environmental limitations this results in the formation of superficial (epiedaphic) and/or subsurface (hypolithic/endedaphic) organogenous horizons of soils. From the perspective of soil science, the superficial varieties of BSC include classical soil litters dominated by mosses and/or lichens, which mostly occur in sheltered environments like wind isolated small valleys or rock “baths” with the meltwater supply from snow patches. Subsurface varieties include hypolithic horizons sometimes extended deeper into endedaphic environment and are dominated by cyanobacteria, green algae, fungi, actinobacteria and more rarely bryophytes. The local conditions provide plenty of spatial and successional transitions between the open and cryptic organogenous horizons and the compositions of communities in them^20^.

Several decades of comprehensive studies on cryptic endolithic communities in hard rocks^21–24^ among the issues of their ecology, diversity and weathering capacity highlighted the endolithic pathway for initial soil formation^25,26^. At the same time, the soil-forming role of hypoliths, which represent another type of cryptic communities, is not well understood. Although hypolithic organisms often produce the only organogenous horizon that is recognized in a soil profile, their influence on soil development in the ice-free landscapes of East Antarctica is barely documented at a detailed scale.

Hypolithic communities (hypolithons) themselves, whether perceived as BSC or not, have been recognized in a number of works^27–29^ and received much attention in terms of their biological features like diversity, composition and also ecology, especially escape and adaptations to the harsh environment. Hypolithons may create “hot spots” of primary productivity, rise bioavailability of nitrogen, act as a refuge source for further dispersal of organisms across various soil environments and eventually provide an important basis for the survival of depauperate ecosystems^28,30–34^. Once moisture is available hypolithic communities can develop quite rapidly^33^, extend into soil and stabilize it like other BSC by cyanobacterial and algal EPS, fungal and actinobacterial mycelium, as well as rhizoids when mosses are involved.

The field studies in East Antarctica (Dry Valleys) reported on the three major types of hypolithons distinguished upon their composition and morphology: cyanobacteria-dominated (type I), fungus-dominated (type II) and moss-dominated (type III)^20,35^. Deeper extension of cryptic communities into soils under pavements could still be referred to hypolithons^33^, or regarded as a separate phenomenon – endedaphic community^36^. We adopt here that hypolithic systems could have several levels of organization: (1) biofilms directly attached to undersides of the pavement components; (2) deeper extension of biomass into sandy bedding of the pavement dominated by cyanobacteria, algae and/or fungi and actinobacteria, as well as moss embedded in the soil matrix under pavement; (3) dead biomass and products of biota-to-mineral interactions both on the undersides of pavement components and in the sandy bedding. Further in the paper we denote these three structural levels collectively as hypolithic horizon of the soil, although each level can have a different degree of manifestation depending on the local ecological conditions.

In the Larsemann Hills, various components of terrestrial ecosystems have been previously investigated including the content of macro and trace elements in soils, sediments and lakes^37–42^. Biological properties of soils and the soil microbial communities were also assessed^43–47^ and the cryptic subsurface communities were described in lithosols^48^. However, we are not aware of extensive research on these hidden soil habitats in the Larsemann Hills and specifically on their organic matter reservoirs. The objective of this study is to provide a detailed scale data on cryptic organogenous horizons in soils of the Larsemann Hills and to evaluate their contribution to the soil organic matter buildup, as well as to raising complexity of the soil cover in a depauperate landscape. The major focus is given to the hypolithic horizon, which we hypothesize provides additional and measurable input to the formation of topsoil. This study is also aimed to give a georeferenced snapshot of the hypolithic BSC and adherent soil horizons that could serve as a local-scale baseline to trace possible future alterations of hypolithic communities in East Antarctica induced by the climate change.

## Materials and Methods

### Study area

The survey was conducted in the Larsemann Hills of the Princess Elizabeth Land (Prydz Bay, East Antarctica) which is a ~50 km^2^ ice-free hilly terrain (oasis). The parent rocks are composed of predominant heterogeneous paragneiss, layered cordierite-gneiss complexes and leucogneiss^49^ and are deeply dissected by the short (<1 km) valleys formed by glacial erosion along lineaments^50^. The glacial deposits are represented mainly by gravelly sands with a low content of clay and silt^44^ and are often covered by the gravel (desert) pavements. The inter-hill valleys extend up to 25% of the total ice-free area and serve as spots of increased biodiversity and productivity as their floors receive meltwater from the perennial snow accumulations^51^. Radiocarbon dating of lake sediments suggests^52^ the territory was partly ice-free since at least 44 ka BP with longer ages (~100 ka) of the local ice-free history evidenced by the surface exposure dating with cosmogenic nuclides, and the rock weathering rates^50^.

The climate of the coastal ice-free areas at the Princess Elizabeth Land is much milder than in the Dry Valleys, and the Larsemann Hills is one the warmest area in East Antarctica with the mean annual air temperature (MAAT) −9.8 °С. The mean air temperature in January is +0.6 °С, but the daytime temperatures may rise to +10 °С^53^. The soil surfaces experience higher temperatures and longer periods with T > 0 °C than the aboveground air with a maximum of +13.9 °C recorded directly under the gravel pavement and +6 °C at 20 cm depth at the same time^51^. This gradient suggests rather favorable temperature conditions that could shortly occur in the hypolithic environment and underlying soil horizons. Although mean annual wind speed is 6.7 m s^−1^, the strong katabatic winds may accelerate up to 50 m s^−1^, which is critical for the retention of BSC on the surface. Annual snow precipitation reaches 250 mm liquid equivalent with no continuous snow cover established. During austral summer the soils at the valley floors are supplied by water due to rare snowfalls, melting of snow patches and subsoil ice schlieren. The suprapermafrost moisture reservoir is poorly available for the surface/subsurface BSC because of the low capillary rise in the gravelly sands, while the thaw depth could reach 61–85 cm^54^. The patterned ground is common on floors and low flanks of the valleys.

### Samples collection

**Figure 1:**
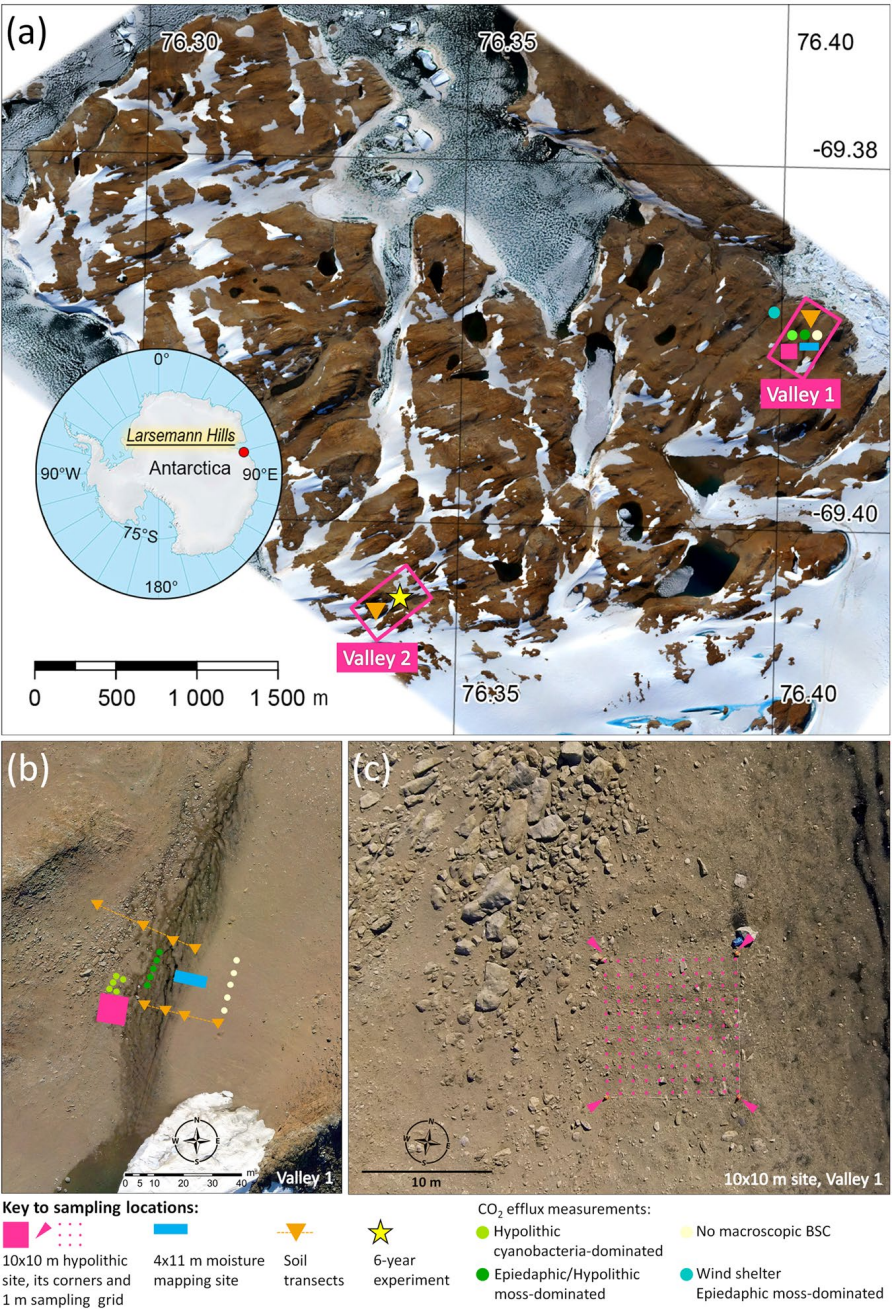
The study sites in the Larsemann Hills, East Antarctica: (**a**) location of the two key valleys at the orthophotomap of the Brokness Peninsula obtained from the mosaic of UAV images as of January 29, 2017; (**b**) orthophoto of the valley 1 with sampling locations; (**c**) close view on the 10 × 10 m hypolithic site from helicopter at the day of sampling (January 29, 2016).

The data presented in this paper was obtained at the two key sites in the Larsemann Hills (Fig. 1a and Fig. S1). One is a coastal inter-hill valley (valley 1; 69.3901°S, 76.4039°E), which is ~400 m long and ~150 m wide, and the floor is supplied by meltwater from the ~5000 m^2^ snow patch traceable on the Google Earth images at least between 2004 and 2018. This key site represents the common pattern of hypolithic colonization in the Larsemann Hills. The second key site is located in one of the most fertile and vast glacial valleys of the Larsemann Hills (valley 2; 69.4042°S, 76.3431°E), and was selected for its extensive and diverse BSC including hypolithic, epiedaphic and amphibian series. The soil samples were harvested during the Russian Antarctic Expeditions in January–March 2010 and January 2016, while the aerial photo data was collected in January 2016 from the helicopter and in January 2017 from the unmanned aerial vehicle (UAV, see section below for details). Various types of organogenous horizons with the focus on hypolithic varieties, their thickness, moisture content, TOC and TN content, as well as TOC/TN ratios were studied at a detailed scale at several sites (Fig. 1a,b,c and Fig. S2): along the 10 × 10 m grid with a step of 1 m and 121 sampling points (“10 × 10 m hypolithic site”, valley 1), along the 4 × 11 m grid with a step of 1 m and 60 sampling points (“4 × 11 m moisture mapping site”, valley 1), two transects in the valley 1 (four pits each), four core pits specific to various types of BSC in the valley 2. Samples (180 totally) were collected by hands. Material of hypolithic horizons was taken immediately under the gravel pavement and between its components: it was detached and scrapped from the undersides of gravels when possible; if colonized the sandy bedding of the pavement was also sampled. Depending on the amount of material samples varied in weight (~1–50 g). Silicone gloves were used to prevent contamination significant to a substrate with low organic matter content. Samples were stored frozen (including transportation period on the vessel) until they were brought to the lab, air-dried or freeze-dried depending on the purpose of further experiment.

### Meso- and micromorphology

We used optical microscopy and scanning electron microscopy (SEM) as a tool to observe biocrust-to-mineral assemblages in selected samples from hypolithic horizons. Morphology of samples was studied on different hierarchical levels - under Leica MZ6 binocular and Nikon Eclipse E200 optical microscope all with digital cameras. The samples were observed in native state and reflected light. SEM was performed in secondary electrons mode at JEOL JSM-6610LV (Institute of Geography RAS, Moscow, Russia) and was combined with the energy-dispersive X-ray spectroscopy (EDX, Oxford Instruments) for elemental analyses.

### Soil organic matter

The content of total organic carbon (TOC), total nitrogen (TN) and carbon stable isotope ratio (δ^13^C) in soil samples (<2 mm) were determined using Vario ISOTOPE Cube CHNS-analyzer coupled for measurements of stable isotope ratios with Isoprime PrecisION IRMS (Elementar, Germany / Ionplus, Switzerland). Prior to measurements, inorganic C was removed with 1 M HCl treatment and then rinsing in deionized water. Living parts of mosses when present were selected on the sieve and removed so that only dead material (mostly peat) was considered in further calculations. Samples were dried at Scientz-10N freeze dryer and crushed for homogenization in Retsch Mixer Mill MM 400. TOC density (g C m^−2^) in organogenous soil horizons was estimated upon TOC concentration, horizon thickness, fine earth (<2 mm) to coarse fragments (>2 mm) ratio and bulk density. The latter was assessed in 141 cm^3^ monoliths dried to a constant mass and weighed. Organic carbon reservoirs of different organogenous horizons formed with participation of hypolithic BSC were calculated by multiplying their TOC densities and areas that they occupy at the floors of the two key site valleys.

Due to a small size of the study objects (especially samples from cyanobacterial hypolithic horizons) and often low concentration of TOC the ^14^C dating required modified techniques of sample preparation including mild acid treatment, which are described in Zazovskaya *et al*.^55^. Graphitization and pressing of targets for ^14^C AMS were conducted in the Radiocarbon Laboratory of the Institute of Geography RAS (Moscow, Russia) with the automated graphitization system AGE 3 (Ionplus, Switzerland). ^14^C AMS measurements were performed at the Center for Applied Isotope Studies, University of Georgia (Athens, USA). The radiocarbon dates were calibrated in OxCal v.4.3.2^56^ (https://c14.arch.ox.ac.uk/oxcal.html), using SHCal13^57^ and Bomb13SH12 atmospheric curves^58^.

### Mapping of hypolithic horizons and BSC

The data on TOC and TN content, as well as TOC/TN ratios were spatially processed for the 10 × 10 m hypolithic site. To interpolate datasets of discrete points to a spatially continuous surface we used ordinary kriging, which is a well-established and common geostatistical method for this purpose^59,60^. The obtained 121 point dataset was not linearly distributed and the grid file had a skewed distribution with some distant outliers from the bulk of the data. To address this issue, we used the equal area level method that allows calculation of contour levels so that there are an equal number of grid nodes within each contour interval. It avoided the situation when most of the contour lines are concentrated around the areas of most dramatic change in the element content, and the details in the smaller variations of TOC, TN and TOC/TN are lost. At each sampling point the microtopography was marked either as polygon or trough.

To extrapolate the field data we used the high resolution visible imagery from UAV. The total absence of a masking vascular plant cover made the color/brightness parameters and morphological characteristics of the soil surface largely informative on the presence of different BSC varieties as well as topsoil moisture conditions and resulted in hypothesis that the optical parameters of topsoil and BSC observed in the field could be attributed to their optical signatures in the visible spectrum observed on the UAV orthoimages. The UAV flight was performed at 250–300 m height on January 29, 2017 by GeoScan 201 complex (Geoscan Group of Companies, Russia, https://www.geoscan.aero/en) equipped with a 35 mm Sony DSC-RX1 camera (Sony Corporation, Japan). We utilized Agisoft Metashape Professional 1.5.4 software package (https://www.agisoft.com) for the photogrammetric processing. Orthophotomosaic with 6.7 cm/pixel spatial resolution, digital elevation model (DEM) with 13.4 cm/pixel spatial resolution and also point cloud (with elevation and RGB color attributes) were produced and processed for morphometric spatial analysis and further mapping. Although georeferenced RGB orthophotomosaic was obtained for the whole area of the Larsemann Hills, for this study we employ the data only on the regions with the most abundant hypolithic BSC at the two key valleys of the Brokness Peninsula (valley 1 – 17 391 m^2^ and valley 2 – 56 232 m^2^). Images clustering, expert interpretation and subsequent mapping were carried out in QGIS environment: QGIS software package v3.4 (https://qgis.org/en/site) with OrfeoToolBox (OTB) plugin for mapping and image processing. DEM and point cloud profiling were performed in the Global Mapper v20 package (https://www.bluemarblegeo.com/products/global-mapper-download.php). The method of unsupervised K-means classification was employed for image processing. The choice in favor of unsupervised classification was made due to the uneven brightness characteristics within RGB orthomosaic (although corrected for brightness) that were inherited from the changes in imaging conditions. The classification was performed in 10 iterations and there were more than 10 000 closed contours produced for each valley. Such high numbers demonstrated the complex pattern of various BSC and barren soil surfaces that interchanged at a very close distance. It was established by experience that it is sufficient to allocate a maximum of 12 classes within a fragment of an image related to the key site to cover the diversity of all BSC types and also barren soil surfaces that lack macroscopic BSC, consolidated rock exposures and debris, snow patches and meltwater. Since BSC distribution had a close connection with the slope angles we also used detailed profiling combined with the DEM-based slope maps to control cluster-to-class attribution upon the morphometric parameters. Assessment of the classification quality was based on the field descriptions, field photographs, aerial photographs from the helicopter on the day of sampling and morphometric characteristics of the meso- and microtopography. For the vast valley 2, we employed additional field observations upon 50 × 50 m grid with a step of 5 m and 121 observation points (“50 × 50 m observation grid”, Fig. S2); no samples were taken and the grid was used only to record the types of BSC. Manual correction of classes and contours obtained from image classification aimed to get the best fit to the field-observed optical and morphological parameters of BSC resulted in totally 6 BSC classes distinguished in the valley 1 and 5 BSC classes in the valley 2. Further BSC mapping was carried out on the basis of obtained classification and the areas of each BSC class were calculated for each valley separately.

### Moisture content mapping

Samples were harvested at the 4 × 11 m moisture mapping site in the valley 1 (69.39004°S, 76.40427°E; grid with a step of 1 m and 60 sampling points). Although being compact the grid has captured all major types of BSC and associated soils in the valley and transected zones with ahumic^61^ soils completely lacking macroscopic organogenous horizons, soils with hypolithic horizons, soils with epiedaphic horizons and soils with amphibian biofilms (develop in subaquatic and partially subaerial conditions due to instability of meltwater supply). The moisture content was determined by the gravimetric method. Samples were collected by hands from the 5 cm top layer, weighted in the field lab, transferred to the stationary lab, dried at 105 °C and weighted. When the gravel pavement was present the samples were taken directly under it in the sandy bedding. In the case of epiedaphic moss-dominated horizons, the moisture content was measured in peat and mineral fine earth immediately beneath the living biomass. The ordinary kriging was used to interpolate dataset from discrete points to a spatially continuous surface, and the equal area level method was employed to produce the contour map.

### CO_2_ efflux

The CO_2_ emission from soils with different organogenous horizons (Fig. 1) was measured in the field by the direct-flow method in closed chambers with portable infrared СО_2_-analyzer AZ 77532 Temp. CO_2_ (Taiwan) calibrated upon Li-8100А (LiCor, USA), which in turn was calibrated using gas standards. We used cylindrical PVC opaque chambers with an area of 90 cm^2^ and a volume of 1–2 liters. Living top parts of mosses were removed if present. Before the measurements open chambers were inserted into the soil at 2–3 cm depth and left for 2 hours, then tightly closed with a lid connected by cellulose tubes to СО_2_-analyzer. Measurements were conducted for 3–6 min depending on the flux rate. 10 repetitions per site were applied. Built-in fans were used to mix the air inside the chambers. Volumetric moisture content in the topmost soil layer necessary for the calculation of CO_2_ fluxes was measured by ML3 ThetaKit with HH2 Moisture Meter and ML3 ThetaProbe Soil Moisture Sensor (Delta-T, UK) and controlled in part by the gravimetric water content. The air and soil temperatures were estimated by the digital thermometer Checktemp-1 (Hanna Instruments, USA) with a stainless steel probe.

### 6-year field experiment: exposure of sterile glass slides in hypolithic environment

To study the development of microorganisms in hypolithic horizons *in situ* the sterile glass slides (75 × 25 mm) were installed into soil immediately under the gravel pavement in March 2010 and stayed there for 6 years until excavated in March 2016 (Fig. S2). This method is based on the ability of microbial cells to develop and overgrow on quartz glass in the attached state at the interface of the liquid and solid phases, where the concentration of substances in the soil solution is increased due to adsorption. In the hypolithic environment, this could partially imitate attachment of microorganisms to the translucent quartz grains and inform about the patterns of colonization that establish on the new mineral surfaces. At the end of the exposure period, the glasses were carefully removed from hypolithic horizons with a non-slip motion, frozen until transferred to the lab and then dried. Slides were examined by the light microscopy to reveal microbial “landscapes” that developed on translucent glass material during 6-year exposure in hypolithic environment. Morphological and metric measurements of microbial cells were carried out using the ScopePhoto v3.1 (http://www.scopetek.com/). The volume of cells was calculated by the method described in Hillebrand *et al*.^62^. The volume of fungal mycelium was calculated as a cylinder. The average cell density of microorganisms was taken as 1.1 g cm^−3^ and the dry matter content as 20%. The dry biomass was calculated per cm^2^ of the slide surface.

### Data representation and statistical analysis

The graphs across the manuscript were produced in Plotly Chart Studio (https://chart-studio.plot.ly). Elements of the box plots represent minimum excluding outliers, first quartile, median, third quartile, maximum excluding outliers, while the points represent individual values of the whole data array including outliers. The data in the text is reported as mean ± standard deviation except for the CO_2_ emission data reported as mean ± standard error of the mean. The non-parametric Mann-Whitney U test was employed to assess whether there was a statistically significant difference between medians of hypolithic TOC and TN concentrations on polygons and troughs.

## Results and Discussion

### Hypolithic horizon: types and transitions

**Figure 2:**
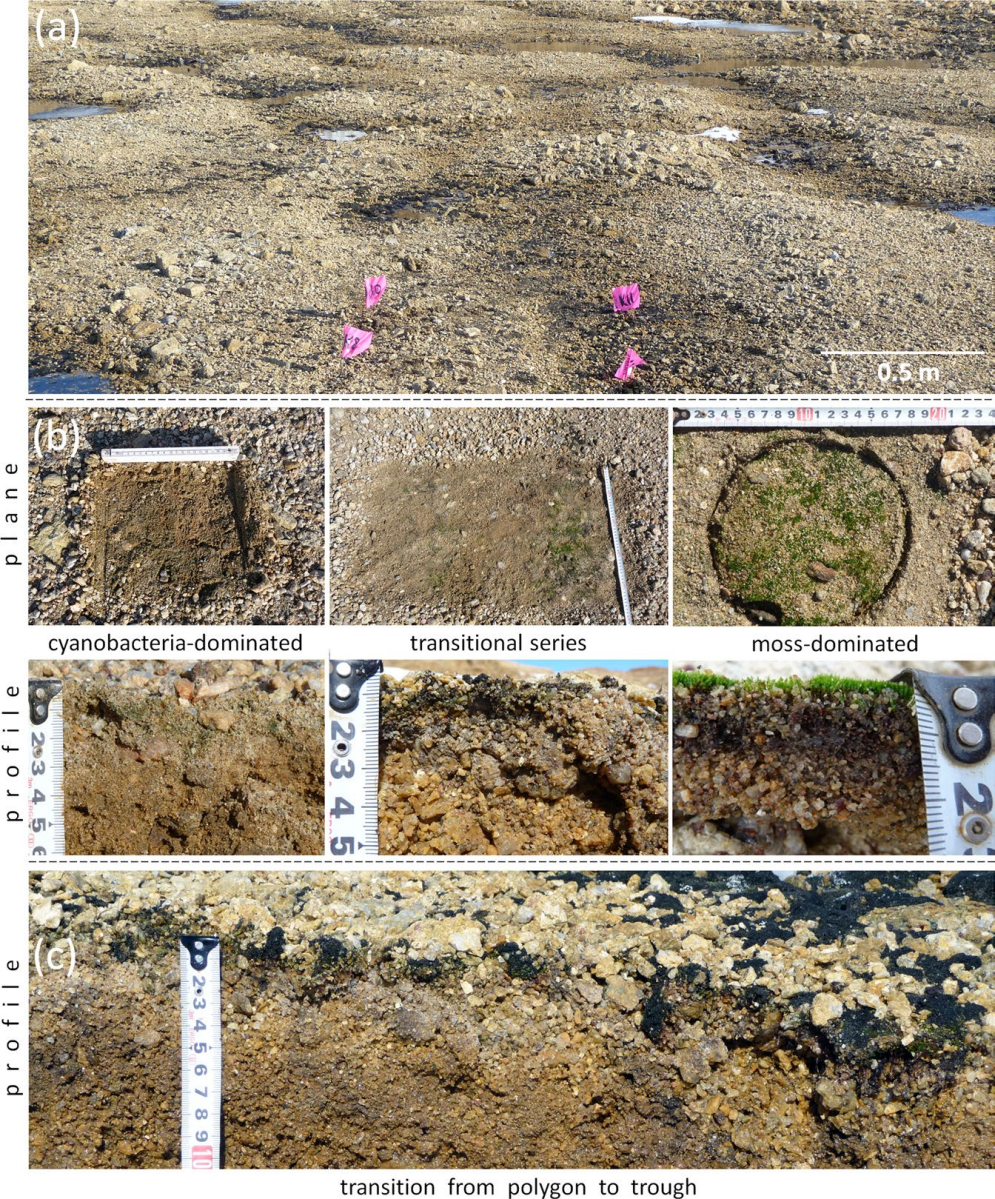
Major types of hypolithic organogenous horizons produced by BSC in soils at the valley floors in the Larsemann Hills: (**a**) patterned ground controls moisture distribution and transitions from cryptic hypolithic organogenous horizons (on polygons) to patchy epiedaphic organogenous horizons (in troughs); (**b**) plane and soil profile view on various types of hypolithic horizons on polygons; (**c**) soil profile in the transitional zone between hypolithic horizon produced by cyanobacteria- and moss-dominated BSC (polygon periphery) to the patchy epiedaphic horizon produced by moss-dominated BSC (trough). The tape-measure on photos is in cm.

**Figure 3:**
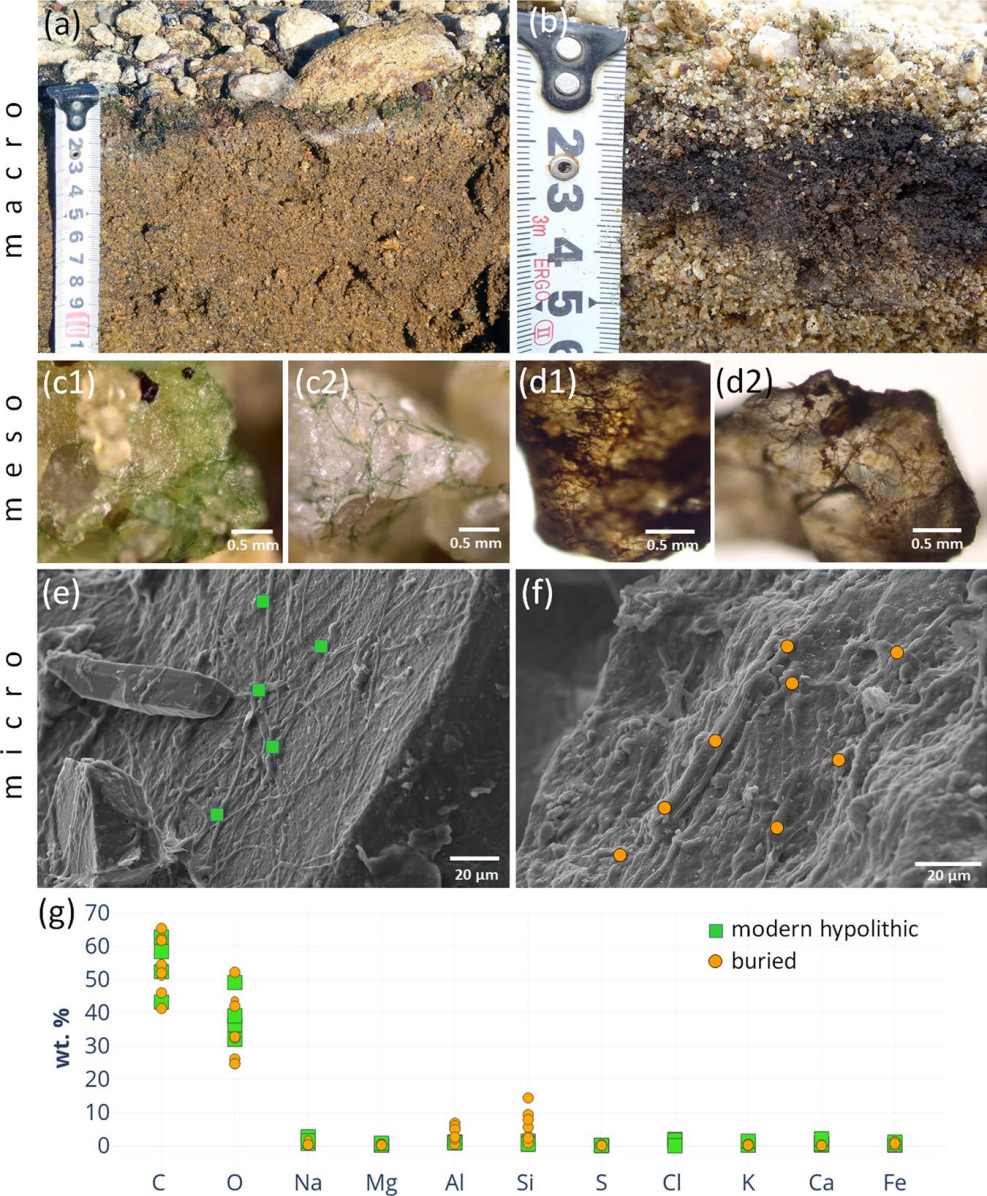
Morphology and composition of cyanobacteria-dominated hypolithic horizons at various scales: (**a**) cyanobacteria-dominated morphotype of the high center polygon at the wet valley floor; (**b**) dark colored sandy material buried under contemporary cyanobacteria-dominated hypolithic horizon in microtopography trap at the polygon periphery; (**c1, c2**) filamentous morphology of cyanobacterial biofilm from the contemporary hypolithic horizon, optical microscopy, reflected light; (**d1**, **d2**) − filamentous morphology of presumably cyanobacterial biofilm from the buried horizon found in microtopography trap at the polygon periphery, optical microscopy, reflected light; (**e**) SEM-SE image of the contemporary cyanobacterial biofilm on the surface of feldspar particle; (**f**) SEM-SE image of the presumably cyanobacterial biofilm on the surface of feldspar particle, buried horizon found in microtopography trap at the polygon periphery; (**g**) elemental composition of modern hypolithic and buried horizon as suggested by the EDX data, orange circles and green squares indicate values obtained from individual EDX measurements, corresponding acquisition sites are marked at SEM-SE images e, f.

Hypolithic horizons most widely occurred in soils of the valley floors and flanks, which were elevated at least ten centimeters above the zone of meltwater streams at the peak of the warm season. We observed two distinct morphotypes of hypolithic horizons (Fig. 2): (1) *cyanobacteria-dominated*, which comprised biofilm-covered sandy particles several centimeters beneath the gravel pavement and patchy biofilms on the undersides of pavement components and (2) *moss-dominated*, which comprised living moss biomass beneath the pavement and peat material below it (rarely thicker than 5 cm). We also observed *transitional series* of hypolithic horizon, that were represented by both cyanobacteria- and moss-dominated morphotypes with the latter one proliferating into epiedaphic environment between unevenly distributed pavement components. We did not find a separate fungus-dominated morphotype like in studies of the Dry Valleys soils^20,35^, although fungi mycelium and spores were previously reported from hypolithic habitats of the Larsemann Hills^46^. We also recognized dark colored horizons with filamentous biofilms on sandy particles that were buried in the microtopography traps immediately below contemporary hypolithic horizons dominated by cyanobacteria (Fig. 3).

Occurrence and transitions between the two morphotypes of hypolithic horizons depended on the topography gradients, which redistributed meltwater when available and enabled development of various BSC types. We observed lateral hypolithic patterns both on the scale of tens of centimeters due to cryogenic microtopography (modified by rill erosion near the thalweg zone) and on the scale of several meters due to the mesotopography gradient from the valley floor to its gentle slopes. At the high flanks of the valley, the macroscopic organogenous horizons (even the cryptic ones) were almost absent giving a way to the barren ahumic soils with no visible traces of photoautotrophs. At the valleys floors, the patterned ground often controlled moisture distribution and transitions from cryptic hypolithic organogenous horizons on polygons to patchy epiedaphic organogenous horizons in troughs (Fig. 2a). Polygon periphery was a transitional zone between hypolithic horizon produced by cyanobacteria- and moss-dominated BSC to the patchy epiedaphic horizon produced by moss-dominated BSC in trough (Fig. 2b,c).

**Figure 4:**
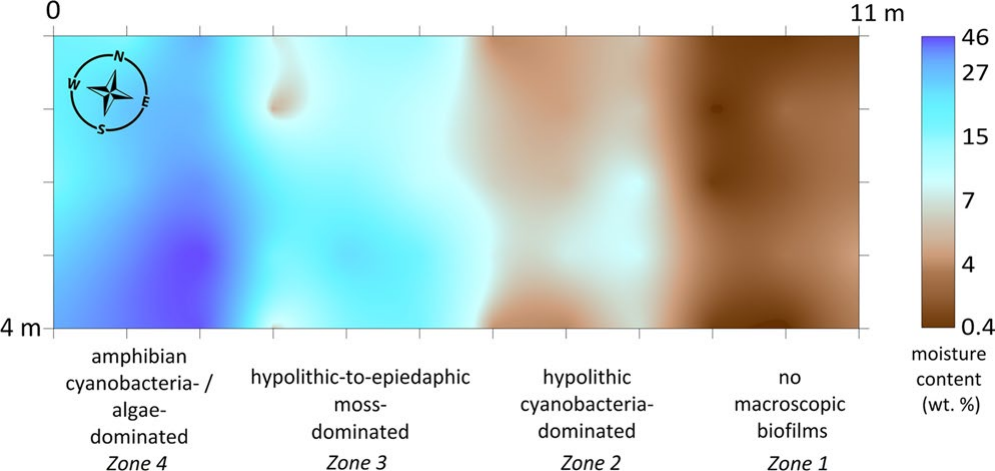
Spatial distribution of gravimetric moisture content (top 5 cm of soil) and different types of BSC at the 4 × 11 m moisture mapping site (60 measuring points) in the valley 1 of the Larsemann Hills (69.39004°S, 76.40427°E; for location in the valley see also Fig. 1). Note: (1) when the gravel pavement was present, the samples were collected directly beneath it in the sandy bedding; (2) in case of epiedaphic moss-dominated horizons the moisture content was measured in peat and mineral fine earth immediately beneath the living biomass.

We determined a lateral gradient in the gravimetric moisture content in the top 5 cm of soil at the 4 × 11 m moisture mapping site in the valley 1. This gradient arose from the 2 m difference in elevation between the valley floor and its east-southeast flank (Fig. S5c) that caused meltwater redistribution. We found that observed BSC types occurred within different moisture ranges in 4 zones (Fig. 4). There were no macroscopic biofilms found on the undersides of pavement components and in its sandy bedding in a range of 0.4–3.7 wt. % of moisture (*zone* 1). Hypolithic cyanobacteria-dominated morphotype was present when moisture content varied between 3.8–7.8 wt. % (*zone* 2). The first moss-dominated hypolithic horizons scarcely appeared when soil moisture content approached 7.0 wt. %, then became continuous and mixed with epiedaphic moss-dominated horizons with up to 27.1 wt. % of soil moisture (*zone* 3). Tongues of amphibian cyanobacteria- and algae-dominated BSC appeared in a range of 18.7–46.1 wt. % (*zone 4*). The cryogenic microtopography (slightly modified by rill erosion) in *zone 2* and partially in *zone 3* caused the interplay of the two moisture ranges: 3.8–7.8 wt. % that was correspondent to the cyanobacteria-dominated hypolithic horizons on polygons and 7.0–17.0 wt. % that was common to the transitional series and moss-dominated horizons in troughs. It is well established that hypolithic colonization is highly correlated with water availability at the global scale^63^. The gradients in moisture content estimated in this case study from the Larsemann Hills also gave a very good explanation to the observed BSC sequences and associated carbon-rich horizons both on the micro- and mesotopography levels. At the same time, we are aware that caution is needed in attributing the field measured moisture content directly to the physiological processes in BSC and the soil organic matter build-up since it can vary a lot in time. Previously Ellis-Evans^48^ found subsurface communities were still present in lithosols of the Larsemann Hills within 1–2 wt. % of soil moisture and the laboratory experiments with cyanobacterial hypoliths of hot deserts showed that primary production required a minimum of 15 wt. % of soil moisture^31^.

According to the field observations in 2010 and 2016, the patterned ground at the wet valley floors had a quasistationary surface. Along with cryogenic translocation of material due to the formation and decay of polygons, there were clear signs of the lateral transport of fine earth at the microtopography scale from the high centers to the more wind-sheltered troughs. We hypothesize that these processes enabled deeper burial of organogenous material, which was originally produced in hypolithic/epiedaphic environment, and therefore led to the enrichment of soil in organic matter. We observed the possible long-term consequence of these processes as the formation of dark carbon-enriched layers few centimeters beneath contemporary hypolithic horizons (Fig. 3b). These buried layers consisted of the sand particles enclosed in biofilms. The biofilms had a distinct filamentous morphology (Fig. 3d,f), which was very similar to the morphology of cyanobacterial biofilms (Fig. 3c,e) from the hypolithic horizon above. The elemental composition obtained from EDX spectra was also in the agreement between contemporary and buried layers (Fig. 3g), although Al and Si abundances were higher in the buried material. The buried biofilms were thick (10–15 µm) and the biogenic filaments were embedded in the amorphous material. The thickness of biogenic coating on the sandy particle of more than 10 µm secured that the Al-Si signal extracted by SEM-EDX originated from the coating itself, but not from the mineral background. This indicates biofilm fossilization and possible close integration of organic matter and amorphous Al/Si-containing species that made this biofilm-to-mineral association more stable.

### Organic matter in soils with hypolithic horizons

**Figure 5:**
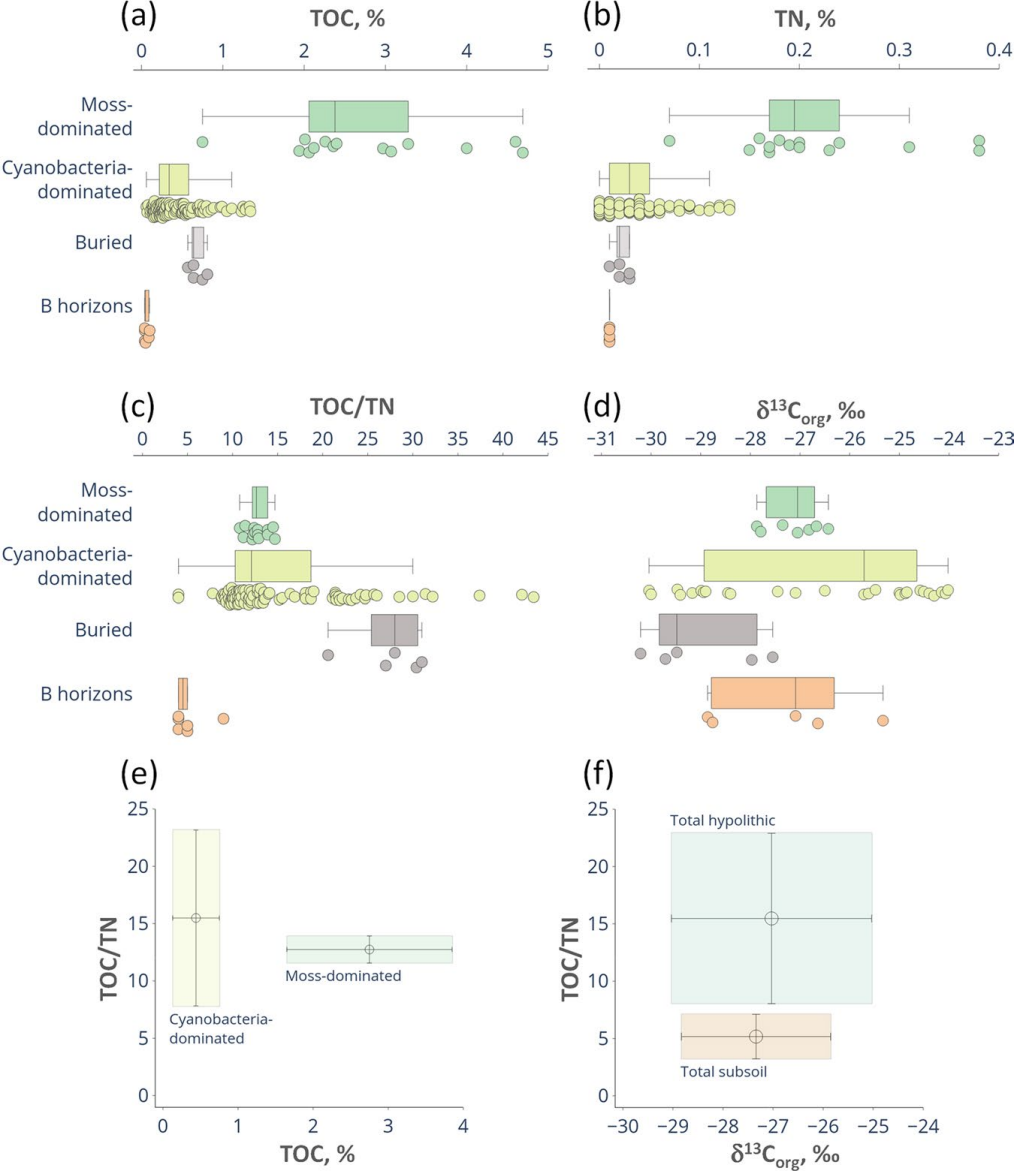
TOC (**a**) and TN (**b**) concentrations, TOC/TN (**c**), carbon stable isotope ratios δ^13^C_org_ (**d**), TOC-TOC/TN (**e**) and δ^13^C_org_-TOC/TN (**f**) plots in various types of soil horizons at the valley floors, Larsemann Hills. Elements of the box plots represent minimum excluding outliers, first quartile, median, third quartile, maximum excluding outliers, while the points represent individual values of the whole data array including outliers. The values plotted at (**e**) and (**f**) are the means of δ^13^C_org_ and TOC/TN values and the bars are standard deviations.

A set of 180 samples was processed to obtain the total organic carbon (TOC) and the total nitrogen (TN) concentrations, as well as TOC/TN ratios in soils with hypolithic horizons located at the wet valley floors in the Larsemann Hills. The whole data array revealed that TOC concentrations were never <0.04% with a mean of 0.68 ± 0.85% (±1 standard deviation) and TN concentrations ranged from 0 to 0.38% with a mean of 0.05 ± 0.07%, indicating that values were widely spread out. The lowest values were recorded in barren ahumic soils with no macroscopic traces of BSC (even in the cryptic niches), which contained 0.04–0.07% TOC and 0.01–0.02% TN or no detected nitrogen at all (Table S1). At a detailed scale there were distinct differences in concentrations of carbon, nitrogen and their ratios within hypolithic horizons themselves, and also between hypolithic, buried organogenous and subsoil horizons. Moss-dominated horizons expectedly had higher TOC and TN concentrations than cyanobacteria-dominated ones (Fig. 5a,b; Table S2): 2.75 ± 1.10% vs. 0.44 ± 0.31% for TOC and 0.22 ± 0.09% vs. 0.04 ± 0.03% for TN, respectively. The mean of TOC/TN ratio for the whole data array (excluding the cases when nitrogen has not been detected) was 14.9 ± 7.1. The TOC/TN ratio in the moss-dominated horizons was rather low with a mean of 12.7 ± 1.2 and a narrow range of 10.8–14.7, which could be explained by the presence of the well-decomposed and homogenized organic matter, mainly represented by peat with hardly discernible or no plant structure. The TOC/TN values in the cyanobacteria-dominated horizons varied between 4.0 and 43.4 with a mean of 15.3 ± 7.5 and demonstrated a skewed distribution with some distant outliers from the bulk of the data (Fig. 5c). TOC/TN ratios can be misleading if the organic matter content is low and the stoichiometry patterns are controlled by unknown variables, e.g., the legacy carbon or mineral impurities. However, when the cyanobacteria-dominated BSC measurably contributes to the soil organic matter (up to 1.34% TOC in our dataset) the elevated TOC/TN values could indicate the extensive production of the C-rich extracellular polysaccharides^64^ common to the subaerial cyanobacterial biofilms^65^ especially in the extreme environments. The organic matter properties of the moss- and cyanobacteria-dominated horizons are well discriminated on the TOC–TOC/TN plot (Fig. 5e): the moss-dominated series are enriched in TOC while the TOC/TN range is consistent.

Previously, the imbalanced stoichiometry (low C, high N) with a very little biotic control over bulk element ratios was suggested as a general property of soils in extreme environments of Victoria Land in Antarctica^66^. Our findings in the milder setting of the Larsemann Hills suggest a more pronounced biotic influence on TOC, TN and their ratio at least in the hypolithic horizons dominated by mosses and cyanobacteria.

The δ^13^C_org_ values (n = 44) ranged from −30.2 to −24.0‰ (Fig. 5d) with the mean of −27.1 ± 1.9‰ The δ^13^C_org_ interval (mean ± 1 standard deviation) of the subsoil horizons stayed within the total interval of the topsoil hypolithic horizons and highlighted the possible link between hypolithic environment and deeper layers, while the intervals of TOC/TN ratios were clearly discriminated between hypolithic and subsoil horizons (Fig. 5f). Observed δ^13^C_org_ ranges are consistent with the subaerial biogenic sources of organic matter^7^ and this relates not only to the topsoil but also to the subsoil. Our dataset is in line with the δ^13^C_org_ ranges of the endolith-derived organic matter, lichen and moss communities^3,7,67,68^. We found that isotopic signature of the cyanobacteria-dominated hypolithic series of the Larsemann Hills varied in the wider range (−30.0 – −24.0‰), than for the moss-dominated ones (−27.9 – −26.4‰). The observed δ^13^C_org_ range of hypolithic horizons dominated by cyanobacteria is most close to the one in endolithic systems^3,7^, which are also dominated by cyanobacteria, often in the lichenized form. The occurrence of free-living or lichenized fungal communities in cyanobacteria-dominated hypolithic horizons of the Larsemann Hills is plausible and could explain why these horizons are more ^13^C-depleted than surficial cyanobacterial and algal communities^7,68^. Discrimination during biosynthesis in fungi could result in the lower δ^13^C values since fungi contain more structurally complex biopolymers than cyanobacteria or algae^7^. This is also consistent with the presence of mycelium in hypolithic horizons of the Larsemann Hills^46^, which, however, does not form distinct fungus-dominated morphotype.

Although the contemporary biotic influence on the soil organic carbon and its stable isotopes ratio is well pronounced in the Larsemann Hills, we cannot exclude the input from other sources of organic carbon, both in the hypolithic topsoil and the subsoil, e.g., redistributed organic matter from the paleo (legacy carbon) and modern lake sediments^7,69,70^.

### Spatial patterns of TOC and TN at a detailed scale

**Figure 6:**
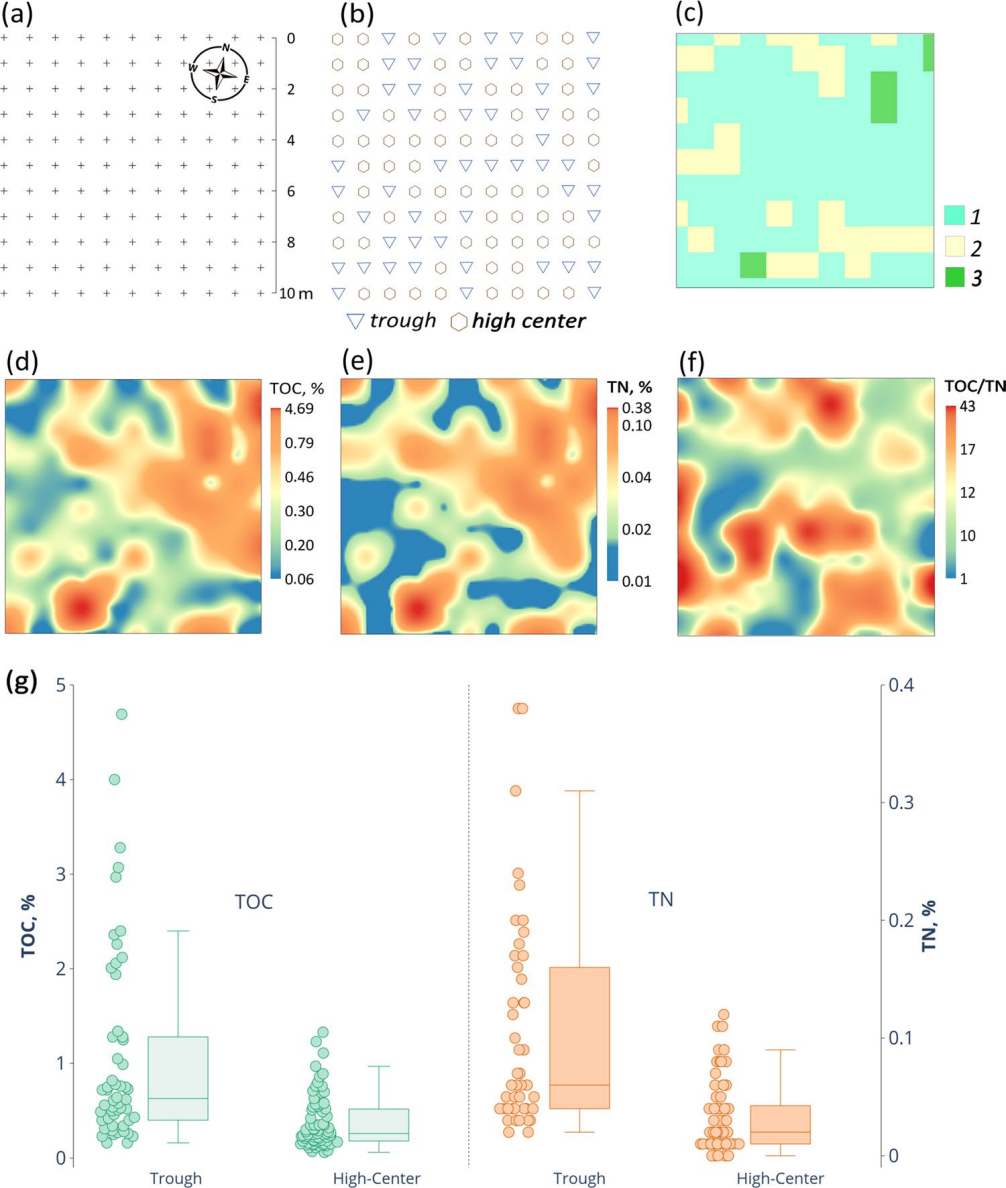
Spatial distribution of TOC, TN and their ratios at a detailed scale (Larsemann Hills, valley 1, 69.3901°S, 76.4039°E): (**a**) sampling design on the 10 × 10 m hypolithic site, crosses mark sampling points; (**b**) microtopography of the key site; (**c**) distribution of hypolithic horizons: *1* – cyanobacteria-dominated morphotype with hypolithic colonization of both the gravel undersides and sandy bedding of the gravel pavement; *2* – cyanobacteria-dominated morphotype with hypolithic colonization of only the gravel undersides; *3* – moss-dominated morphotype with mainly hypolithic colonization and patchy proliferation into epiedaphic environment; (**d**, **e**, **f**) spatial patterns of TOC, TN and TOC/TN, respectively; **(g)** TOC and TN concentrations in hypolithic horizons upon microtopography pattern. Elements of the box plots represent minimum excluding outliers, first quartile, median, third quartile, maximum excluding outliers, while the points represent individual values of the whole data array including outliers.

TOC concentrations at the 10 × 10 m hypolithic site with 121 sampling points ranged from 0.06% to 4.69% and TN values varied between 0.01% and 0.38% (Fig. 6). TOC and TN spatial patterns often correlated and the elements abundances were the lowest in the middle part of the high center polygons, where biofilms were primarily present on the undersides of the gravels and more rarely extended deeper into the sandy bedding. The loci with the highest TOC and TN concentrations were mainly found in the trough areas (Fig. 6g) and were associated with the moss-dominated horizons. TOC values in troughs averaged at 1.07 ± 1.03% and at the high centers – 0.37 ± 0.28%, while TN in troughs averaged at 0.11 ± 0.09% and at the high centers – 0.04 ± 0.03%. Medians of TOC and TN concentrations had statistically significant difference between polygons and troughs (Table S2). A possible explanation of this pattern is that troughs provide better moisture conditions, and also serve as wind shelters at the microtopography level enabling proliferation of hypolithic BSC into the epiedaphic environment or even individual development of the epiedaphic BSC. Analysis of soil temperatures within 20 cm from the surface of the patterned ground showed that the austral summer temperatures in the trough are also more favorable than in the high center of the polygon. The average January temperature at a depth of 20 cm reached + 4.9 °C in trough versus +4.4 °C on high center, while the annual sum of T > 0 °C was 261 °C versus 229 °C, and the annual sum of temperatures >5 °C was 133 °C versus 78 °C, respectively. Increased soil temperatures and longer warm periods in troughs originate from the larger pore space between frost sorted coarse materials in comparison to high centers. The proliferation of mosses into epiedaphic environment in troughs induced by its favorable physical conditions also enables positive feedback on soil temperature regime from better absorption of solar radiation by patches of the dark moss biomass (if patchy moss would develop into continuous epiedaphic cover it could cause the opposite effect due to thermal insulation).

Overall, the detailed mapping at the 10 × 10 m hypolithic site has documented the presence in hypolithic environment of a continuous organogenous horizon that spreads for at least 100 m^2^ and even proliferates from the cryptic series to the open ones upon a patchy pattern. The most common was the hypolithic cyanobacteria-dominated morphotype which consisted of two subtypes: 1) with the colonization of the gravel undersides and 2) with the colonization of both the gravel undersides and the sandy bedding of the pavement. We suggest that troughs which display the highest TOC and TN concentrations are the hot spots of organic matter accumulation at a detailed scale upon a patterned ground template in the presence of meltwater. Potential mechanism of a longer carbon stabilization in troughs involves burial of organic matter by the fine earth eroded from the adjacent polygons or windblown from the more distant sources. This is one of the possible pathways for hypolith-derived organic matter to enrich the soil and produce the buried organogenous layers, e.g. those with dark filamentous biofilms as seen in (Figs. 3b,d1,d2,f).

### Radiocarbon age of TOC

We summarize here the radiocarbon data on TOC from cyanobacteria-dominated hypolithic horizons, moss-dominated hypolithic and epiedaphic horizons, and also subsoil horizons (Table 1 and Fig. S3). Hypolithic and epiedaphic horizons contained a significant amount of modern carbon with the F^14^C(fraction modern)>1 in 4 out of 5 samples from the cyanobacteria-dominated series (max F^14^C = 1.168 ± 0.004), and in 2 out of 3 samples from the moss-dominated ones (max F^14^C = 1.121 ± 0.003). In the hypolithic horizons there were two samples with F^14^C < 1 corresponding to a conventional ^14^C age of 150 ± 20 yr BP (F^14^C = 0.982 ± 0.003) for TOC of cyanobacteria-dominated material and 1230 ± 45 yr BP (F^14^C = 0.858 ± 0.005) for TOC of the moss peat. The shallow B horizon with no macroscopic signs of biota revealed F^14^C > 1 at a depth of 5 cm, which suggests young carbon has leaked to the subsurface, e.g., during the meltwater percolation. The deeper B horizons were as old as 1840 ± 20 yr BP (10–15 cm) and 6690 ± 30 yr BP (20–25 cm) corresponding to F^14^C = 0.795 ± 0.002 and F^14^C = 0.435 ± 0.001. TOC in the shallow buried horizon with fossilized biofilms of presumably cyanobacterial origin (Figs. 3b,d1,d2,f) had ^14^C age of 1105 ± 25 yr BP (F^14^C = 0.872 ± 0.0025).

**Table 1:** ^14^C data for TOC from various topsoil and subsoil horizons in the valley floor biotope of the Larsemann Hills (according to unpublished data and our previous study^55^, the latter is marked with an asterisk; σ – standard deviation).

Lab code	Horizon	Depth, cm	Material	F^14^C (1σ)	^14^C yr BP (1σ)	cal BP (2σ) / CALIBomb AD (2σ)
IGAN_AMS_4945*	Moss-dominated, epiedaphic	0–1	TOC of peat	1.121 ± 0.003	—	1958–1962 cal AD (0.049) 1991–2000 cal AD (0.905)
IGAN_AMS_4946*		1–2	TOC of peat	1.105 ± 0.003	—	1993–2005 cal AD
IGAN_AMS_4944	Moss-dominated, hypolithic	0–1	TOC of peat	0.858 ± 0.005	1230 ± 45	1260–1248 cal BP (0.015) 1245–1218 cal BP (0.021) 1186–970 cal BP (0.917)
IGAN_AMS_4933	Cyanobacteria-dominated, hypolithic	1–2	TOC	1.168 ± 0.004	—	1957–1964 cal AD (0.154) 1985–1995 cal AD (0.800)
IGAN_AMS_4931*		0–1	TOC	1.127 ± 0.003	—	1958–1962 cal AD (0.053) 1990–2000 cal AD (0.901)
IGAN_AMS_5325		1–2	TOC	1.060 ± 0.003	—	1951–1963 cal AD
IGAN_AMS_4930*		0–1	TOC	1.027 ± 0.003	—	1951–1960 cal AD
IGAN_AMS_5326		1–2	TOC	0.982 ± 0.003	150 ± 20	265–221 cal BP (0.218) 148-… cal BP (0.736)
IGAN_AMS_4947*	Buried	2–4	TOC	0.872 ± 0.0025	1105 ± 25	1052–1022 cal BP (0.129) 985–925 cal BP (0.825)
IGAN_AMS_5324	B	4–5	TOC	1.029 ± 0.003	—	1951–1960 cal AD
IGAN_AMS_5323		10–15	TOC	0.795 ± 0.002	1840 ± 20	1814–1768 cal BP (0.190) 1752–1699 cal BP (0.678) 1651–1622 cal BP (0.087)
IGAN_AMS_5322		20–25	TOC	0.435 ± 0.001	6690 ± 30	7585-7460 BP

^14^C data indicates that organic carbon stored in hypolithic environments of the Larsemann Hills is relatively young. This is very different from the findings in the most extreme hyper-arid environments like Atacama Desert, where hypoliths establish long-living communities with ages of up to 12,000 years since the substrate colonization^27^. The water supply is crucial for high productivity of hypolithic communities and their age is positively correlated with aridity^27,33^. In the Larsemann Hills the hypolithic communities of the valley floors are relatively well supplied with meltwater in comparison to the driest locations in Atacama Desert or Dry Valleys. Previously Zazovskaya *et al*.^55^ also proposed the high dynamism of the soil surfaces due to strong winds and occasional erosion by meltwater as a possible explanation of young ^14^C ages of TOC in the topsoil, which one could assume should experience very slow C turnover times in the extreme environments of East Antarctica. Our radiocarbon dataset supports the hypothesis of several time-divergent carbon pools in soils of the Larsemann Hills with rather rapidly cycling carbon decoupled from the ancient one like it was previously proposed for the Dry Valleys soils^4,71^. While the topsoil in the Larsemann Hills has an overwhelming portion of modern TOC there are still 20–30% of cases where organic carbon is stabilized for ~100 years in cyanobacteria-dominated hypolithic horizons and ~1000 years in the moss peat of the epiedaphic/hypolithic organogenous horizons. We report here that the burial under a few centimeters of fine earth is an efficient mechanism to stabilize cyanobacterial biofilms for ages up to ~1000 years. ^14^C age of TOC in deeper horizons of soils points toward at least partial contribution of the legacy carbon.

### CO_2_ efflux from soils

**Figure 7:**
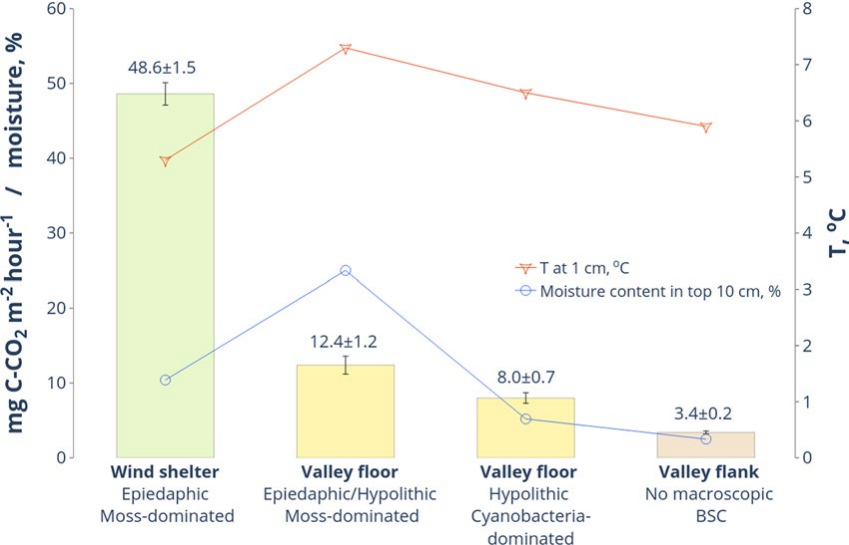
*In situ* measurements of CO_2_ emission (± standard error of the mean) from the surface of soils with various types of organogenous horizons and completely without them.

Carbon dioxide emission from the surface of soils (valley 1, Fig. 7) with the cyanobacteria-dominated hypolithic horizons was as low as 8.0 ± 0.7 mg C-CO_2_ m^−2^ hour^−1^ (± standard error of the mean). The appearance of mosses in the hypolithic environment with scattered proliferation on the day surface slightly shifted emission values to 12.4 ± 1.2 mg C-CO_2_ m^−2^ hour^−1^. This is obviously explained by the higher biomass of mosses, their enhanced moisture capacity and better warm-up of the dark epiedaphic patterns, all together stimulating heterotrophic activity. To better understand CO_2_ fluxes from soils with hypolithic horizons and emplace them on the landscape level we simultaneously measured the surface emission from the most carbon-rich and carbon-poor types of soils in valleys of the Larsemann Hills. We chose soils in the wind-sheltered depressions on the rocky slopes (see Fig. 1 for location) as the most advanced series with the 10 cm thick epiedaphic moss horizons. As the most carbon-poor background control, we employed the barren ahumic soils in the valley 1 with no macroscopic signs of any BSC. The soils in wind shelters had four times higher CO_2_ emission than the moss-dominated mixed hypolithic/epiedaphic series at the valley floors. At the same time, soils with cyanobacteria-dominated hypolithic horizons had less than 5 mg C-CO_2_ m^−2^ hour^−1^ increment in flux in comparison to the dry soils of higher valley flanks, which demonstrated no macroscopic BSC at all. Since the measurements were conducted simultaneously and the temperature and moisture content were comparable, this difference could be indicative of CO_2_ added by cyanobacteria-dominated hypolithic horizons to the CO_2_ flux apart from its abiogenic component. Shanhun *et al*.^72^ showed that biological respiration in the Dry Valleys accounts only for 25% of the measured CO_2_ flux from soil. In the milder environments of the Larsemann Hills, the input from biological respiration could be greater. We cannot completely exclude heterotrophic activity even in the driest (moisture content ~3%) ahumic soils, which contained in our dataset at least 0.04% TOC (Table S1).

### “Microbial landscapes” of hypolithic horizons

**Figure 8:**
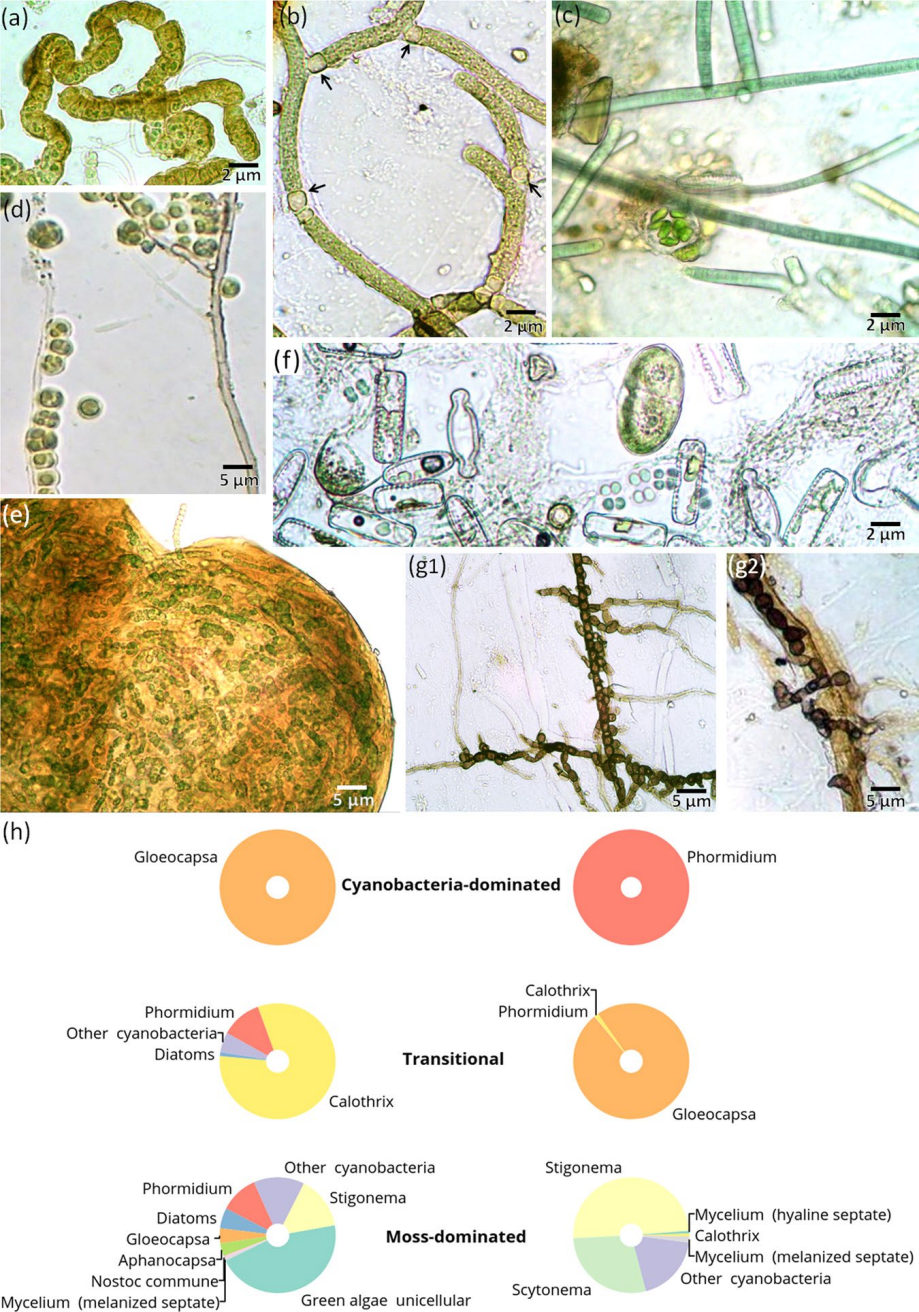
“Microbial landscapes” established on quartz slides after six years of exposure in the hypolithic environment: (**a**) microcolony of cyanobacteria *Stigonema* sp.; (**b**) filamentous cyanobacteria *Scytonema* sp. with nitrogen-fixing heterocysts (black arrows); (**c**) spherical microcolony of coccoid *Chlorophyta* in patchy EPS matrix against the background of *Phormidium* sp. filaments; (**d**) unicellular coccoid algae (*Chlorophyta*) growing along melanized septated fungal hyphae; (**e**) spherical microcolony of cyanobacteria *Nostoc* sp.; (**f**) algae and cyanobacteria association: *Diatomeae*, *Chlorophyta* and small coccoid cells of cyanobacteria; (**g1**) monodomination of the one melanized fungi morphotype with chlamydospore formation and its close association with cyanobacteria (**g2**); (**h**) biomass ratios of various microorganisms that colonized slides during six years of exposure in different types of hypolithic horizons.

The field experiment revealed that microbial hypoliths had colonized intact mineral surfaces over the six-year period. After excavation of glass slides, we observed the “microbial landscapes” that formed in their natural hypolithic environment and consisted of cyanobacteria, unicellular green algae, diatoms, unicellular heterotrophic prokaryotes and fungi (Fig. 8). Colonization replicated the hypolithic pattern with a maximum of microbial biomass attached to the central part of the 75 × 25 mm slides, but not to their upper part, located in vicinity to the day surface. Filamentous cyanobacteria and unicellular green algae prevailed among the primary producers (Fig. 8a–e). Clusters of diatoms have also been detected (Fig. 8f) and can contribute to the primary production as well. However, the established hypolithic complex was dominated by the eurybiontic cyanobacteria (often inhabiting epilithic niche) of those genera, which representatives are also known as lichen photobionts, e.g., *Calothrix*, *Scytonema*, *Nostoc*, *Gloeocapsa*, *Stigonema*, or simply eurybiontic genera, such as *Phormidium*. Cyanobacteria cells often had brown and dark red colors, indicating UV-protecting pigments. Cyanobacteria were predominantly filamentous and produced heterocysts (Fig. 8b) facilitating nitrogen fixation during the polar day and raising its availability in soils. Heterotrophic bacteria and fungi were mainly confined to the loci of primary producers with various forms of their interaction, including parasitism on cyanobacteria, germination of mycelium from lichen soredia, association with algae and cyanobacteria (Figs. 8d,g2). Although the fungal mycelium and spores were abundant as also reported in previous study on soils of the Larsemann Hills^46^ the morphological diversity of fungi was very low with only a few spore morphotypes observed. Specific features included the monodomination of a single fungal morphotype (Fig. 8g1) and the absence of mycelial *Actinobacteria* (actinomycetes). However, we cannot completely exclude the presence of unicellular *Actinobacteria* among the clusters of heterotrophic bacteria that were observed on the slides. In the Larsemann Hills, *Actinobacteria* are the dominant bacterial taxa in soils^44^ and are also found within algae- and cyanobacteria-dominated layers under stone pavements^45^. It is likely that heterotrophs follow the patterns initially created on slides by the primary producers. While the six-year experiment documented intensive colonization of the slides by cyanobacteria and green algae, the heterotrophic colonization may take longer. For instance, *Actinobacteria* in soils of the Larsemann Hills demonstrate significantly higher relative abundances with the longer distance from the glacier^44^, thus longer periods of soil exposure.

Surprisingly the slides were not covered by continuous biofilms that are common on subaerial surfaces including those in extreme environments. Instead of continuous coatings the main biofilm-forming agents (cyanobacteria and green algae) formed discrete and compact spherical microcolonies with abundant EPS (Fig. 8e).

The dry microbial biomass constituted 2.4 ± 0.7 mg per cm^2^ of the slide surface exposed in the cyanobacteria-dominated horizons, 2.3 ± 0.9 mg per cm^2^ in the moss-dominated and 16.3 ± 17.5 mg per cm^2^ in the transitional series. The biomass ratios of various microorganisms that colonized slides were discriminated between cyanobacteria-, moss-dominated and transitional series (Fig. 8h), probably inheriting the structure of microbial communities that was already in place. This is in correspondence to the earlier findings^20,28,35^ on the deterministic processes and successional patterns in hypolithons indicating cyanobacteria-dominated communities as the basal stage. The cyanobacterial hypolithic horizons of the high center polygons produced monodominant pattern of colonization with either *Gloeocapsa* or *Phormidium* as the only taxa developing in attached state on the slide surface. The cyanobacterial diversity increased and diatoms appeared in the transitional hypolithic series, while the highest diversity of cyanobacterial and algal autotrophs, as well as pronounced hyaline and melanized mycelium manifested on slides from the moss-dominated hypolithic horizons.

Thus, the six-year field experiment has shown that the new mineral surfaces are rapidly colonized by the microbial autotrophs within the few years of exposure in hypolithic environment. At this timescale the microbial biomass of colonizers is discriminated upon existing microbial patterns in cyanobacteria-, moss-dominated and transitional series of hypolithic horizons, however, continuous EPS-rich coatings known of subaerial biofilms^65^ do not yet appear.

### Hypolithic horizon at a landscape level

**Figure 9:**
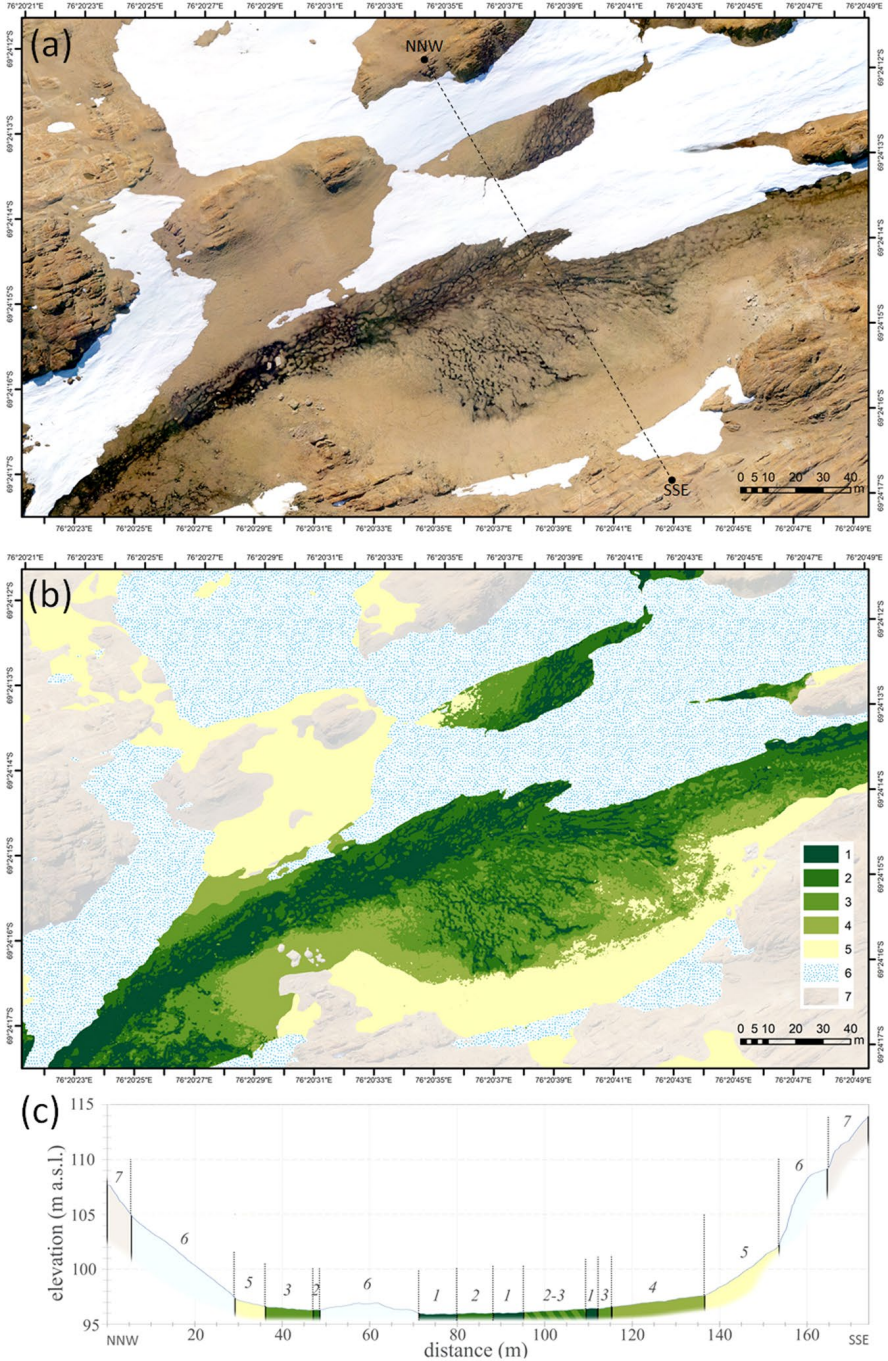
Distribution of BSC in the valley 2 (69.4042°S, 76.3431°E; Larsemann Hills): (**a**) orthophotomap obtained from mosaic of UAV images as of January 29, 2017; (**b**) distribution of BSC classes and other landscape components: 1 – amphibian algae- and cyanobacteria-dominated + epiedaphic moss-dominated (class II); 2 – hypolithic moss-dominated + epiedaphic moss-dominated (class III); 3 – hypolithic cyanobacteria-dominated + hypolithic moss-dominated with patchy proliferation of epiedaphic moss (class IV); 4 – hypolithic cyanobacteria-dominated (class V); 5 – no BSC (barren ahumic soil) or ephemeral BSC (class VI); 6 – snow patches; 7 – consolidated rock exposures and rock debris with epi/endolithic colonization; (**c**) DEM-derived geomorphological profile through the valley as indicated by dashed line on the orthophotomap, the colors and numerals match the legend on (**b**).

In the absence of vascular plant cover, the BSC colonization pattern is clearly recognized on the high-resolution orthoimages in the visible spectrum (Fig. S4) and demonstrates strong connectivity to the soil moisture gradients upon meso- and microtopography of the valleys (Fig. 9 and Fig. S5). We were able to distinguish six classes of BSC on the orthophotomaps, which helped to extrapolate our field data to a landscape level. Hypolithic horizons occurred within three out of six classes of BSC (Table 2): *hypolithic* moss-dominated + epiedaphic moss-dominated (class III), *hypolithic* cyanobacteria-dominated + *hypolithic* moss-dominated with patchy proliferation of epiedaphic moss (class IV), *hypolithic* cyanobacteria-dominated (class V). The stand-alone hypolithic cyanobacteria-dominated BSC (class V) was present in a 0.5–2 m range of elevation over the talweg (Fig. 9c and Fig. S5c) and was emplaced on the valley slopes (3°) as a 2–20 m wide stripe between the lower-slope mixed moss/cyanobacteria classes (slopes < 3°) and the upper-slope barren ahumic soils (slopes 12–20°). It was not possible to discriminate separately hypolithic BSC in the complex patterns of class III and class IV since the hypolithic BSC often occurred at a very close distance to the epiedaphic one. Such patterns could be reliably resolved only upon a detailed sampling as demonstrated previously at the 10 × 10 m hypolithic site in the valley 1. As far as we learned from the field, the barren ahumic soils can occasionally be enhanced by sporadic inclusions of ephemeral hypolithic cyanobacteria-dominated biofilms, although present only on the undersides of the pavement components. This pattern probably originates from colonization after snowfalls when moisture availability shortly rises. However, it could not be spatially resolved by the techniques we used, and therefore was not accounted in further calculations. When all three classes of BSC that contain hypolithic component are combined, they account for 12.0% of the total soil area at the valley 1 and 49.5% of the total soil area at the valley 2, which is well supplied by meltwater and is one of the most fertile across the Larsemann Hills. Cyanobacteria-dominated morphotype of hypolithic horizon occupied 3.0% and 13.2% of the total soil area in the valley 1 and valley 2, respectively. The other two morphotypes of hypolithic horizons (moss-dominated and transitional) could also be attributed to the BSC classes obtained upon classification of UAV orthoimages. Hypolithic moss-dominated morphotype was present in class III and transitional series of hypolithic horizon were most common in class IV (Table 2). We attributed parameters of the three morphotypes of hypolithic horizons to the three most relevant BSC classes to get a rough upper-limit estimate of how much TOC could be stored by various hypolithic horizons across the soils of Valley 1 and Valley 2 (Table 3). Assessment of TOC densities in a triplet of moss-dominated/transitional/cyanobacteria-dominated morphotypes indicated quite an expected decrease of values from the moss- to cyanobacteria-dominated varieties: 429/86/40 g C m^−2^, respectively. Taking into account the maximum areas of corresponding BSC classes, the TOC reservoirs for the moss-dominated/transitional/cyanobacteria-dominated morphotypes comprised 2269/361/136 Kg C, respectively. Approximated TOC reservoir of all horizons formed with a major contribution from hypolithic BSC constituted 2765 Kg C at a 36418 m^2^ of the total soil area in the two valleys. Thus, hypolithic BSC plays at least a distinguishable role in shaping the topsoil and its carbon reservoir at a landscape level, and creates a hypolithic “gateway” for organic carbon to enter depleted soils of the Larsemann Hills.

**Table 2:** BSC distribution in the two valleys of the Larsemann Hills.

BSC class	Morphotype of hypolithic horizon present in BSC class	Valley 1		Valley 2	
		Area, m^2^	Area, %	Area, m^2^	Area, %
I. Amphibian algae- and cyanobacteria-dominated	—	172	1.3	—	—
II. Amphibian algae- and cyanobacteria-dominated + epiedaphic moss-dominated	—	1204	8.7	3487	15.4
III. Hypolithic moss-dominated + epiedaphic moss-dominated	moss-dominated	683	4.9	4606	20.4
IV. Hypolithic cyanobacteria-dominated + hypolithic moss-dominated with patchy proliferation of epiedaphic moss	transitional	568	4.1	3606	15.9
V. Hypolithic cyanobacteria-dominated	cyanobacteria-dominated	408	3.0	2977	13.2
VI. No BSC (barren ahumic soil) or ephemeral BSC	—	10762	78.0	7945	35.1
**Total area of soils**		**13797**	**100.0**	**22621**	**100.0**
**Total hypolithic**		**1659**	**12.0**	**11189**	**49.5**

**Table 3:** Approximation of TOC reservoirs in the topsoil hypolithic horizons across the two valleys in the Larsemann Hills (TOC density and reservoir were calculated from mean values of horizon thickness, bulk density and TOC concentration).

Morphotype of hypolithic horizon	Thickness, cm	Bulk density, g cm^−3^	TOC, %	TOC density, g C m^−2^	Area, m^2^	TOC reservoir, Kg C
Moss-dominated	2.6 ± 1.1	0.6 ± 0.3	2.8 ± 1.1	429	5289	2269
Transitional	1.2 ± 0.8	0.9 ± 0.5	0.8 ± 0.9	86	4174	361
Cyanobacteria-dominated	0.7 ± 0.6	1.3 ± 0.1	0.4 ± 0.3	40	3385	136
**Total**					**12848**	**2765**

### Should the substrate with hypolithic horizon be recognized as a soil

“Hypolithic soils” have been previously recognized in the Atacama Desert by Warren-Rhodes *et al*.^27^. Although this denomination was in quotes, it related to the tiny soil layer adherent to the hypolithic communities dominated by cyanobacteria. The current major classifications stay indifferent to soils with cryptic organogenous horizons. Most of the soils in the Larsemann Hills including those with hypolithic horizons have shallow permafrost or bedrock, thus, are classified as Cryosols or Leptosols in the World Reference Base (WRB) system^73^ and Gelisols or Entisols in the Soil Taxonomy system^74^. The common soil taxa of the valley floors most conductive to the hypolithic colonization are Protic Turbic Cryosols (WRB) or Typic Haploturbels (Soil Taxonomy). However, hypolithic horizons accumulate a measurable amount of organic matter and form a continuous subsurface body as documented in this study. Moreover, hypolithic horizon is located directly inside the soil matrix and, paradoxically, this fact justifies it as a soil horizon in a more distinct way than the epiedaphic moss-lichen litter, which relation to the soil body is sometimes questioned. The presence of hypolithic hotspot of productivity makes the topsoil enriched in biogenic elements and in general raises complexity of the soil cover in depleted environments of East Antarctica.

There is a set of unresolved questions on the role of hypolithic communities in shaping the soil morphology, aggregates formation (when hypoliths extend to the sandy bedding of the pavement), biomineral interactions, elements cycles and long-term stabilization of organic carbon. It is the future task to explore spatial patterns of hypolithic horizons across the ice-free areas of East Antarctica using such proxy as hyperspectral and multispectral UAV imaging combined with the field sampling. Accurate experiments that could document a link to the deeper soil horizons have yet to be established. Hypoliths are highly dependent on the moisture content and their migrational ability is one of the specific features to explore since some cyanobacteria can actively move to the soil surface or retreat to the refuge below in response to the wetting/drying events^75^. The contribution of the hypolith-derived organic matter to the soil and landscapes reservoirs should be quantitatively evaluated and related to the other known sources. Having received answers to these questions, we can better understand whether the phenomenon of hypolithic soils is justified and appropriate proposals for soil classification are required.

## Conclusion

Development of microbial and cryptogamic photoautotrophs in hypolithic habitats of the Larsemann Hills resulted in formation of the subsurface organogenous horizons and substantial organic matter accumulation in the topsoil (up to 4.69% TOC and 0.38% TN). When patterned ground was present, the troughs displayed the highest TOC and TN concentrations in comparison to the polygons, thus, acting as hot spots of organic matter accumulation at a microtopography scale. The detailed mapping has evidenced the presence of a continuous organogenous horizon in the hypolithic environment that spreads for a documented cluster of 100 m^2^, shifts between cyanobacteria- and moss-dominated morphotypes and proliferates from cryptic series to the open ones upon a patchy pattern. At the landscape level, various hypolithic BSC occupied 12.0–49.5% of the total soil area at the floors and flanks of the two studied valleys. The isotopic signatures (δ^13^C_org_ ranges) of subsoil horizons overlapped with the signatures of topsoil hypolithic horizons suggesting that additional contribution of hypolithic carbon to a deeper soil should be accounted along with the other biogenic sources. The field experiment has shown that the new mineral surfaces were colonized by microbial hypoliths within a few years of exposure indicating the fast hypolithic pathway for fresh carbon to enter the soil. The radiocarbon dataset suggests the major part of organic carbon from hypolithic horizons is modern or has a centenary age. In the microtopography traps (e.g. troughs), organic carbon could be stabilized on a millennial timescale through shallow burial by the fine earth eroded from adjacent polygons or more distant locations. This suggests a pathway for the enrichment of depleted subsoil by the hypolith-derived organic matter. The total hypolithic carbon reservoir is superimposed on the subsoil organic matter reservoir that has older age and various origins.

## Supplementary information

Supplementary information.

## Data Availability

The authors declare that the data supporting the findings of this study are available in the article and its supplementary part. Additional datasets generated and/or analyzed during the current study are available from the corresponding author on reasonable request.
