# Supplementary material for: Hypolithic communities shape soils and organic matter reservoirs in the ice-free landscapes of East Antarctica
Source: Sci Rep. 2020 Jun 24;10:10277. doi: 10.1038/s41598-020-67248-3 (PMC7314805; doi:10.1038/s41598-020-67248-3)
Supplement: Supplementary file 1 — Supplementary information. [file 41598_2020_67248_MOESM1_ESM.pdf]

Supplementary information

Hypolithic communities shape soils and organic matter reservoirs in the ice-free landscapes of East Antarctica

Nikita Mergelov[1,*], Andrey Dolgikh[1], Ilya Shorkunov[1], Elya Zazovskaya[1], Vera Soina[2], Andrey Yakushev[2], Dmitry Fedorov-Davydov[3], Sergey Pryakhin[4] and Alexander Dobryansky[1]

[1]Institute of Geography, Russian Academy of Sciences, 119017, Moscow, Russia
[2]Faculty of Soil Science, Moscow State University, 119991, Moscow, Russia
[3]Institute of Physicochemical and Biological Problems in Soil Science, Russian Academy of Sciences, 142290, Pushchino, Russia
[4]Arctic and Antarctic Research Institute, 199397, Saint Petersburg, Russia
*email: mergelov@igras.ru

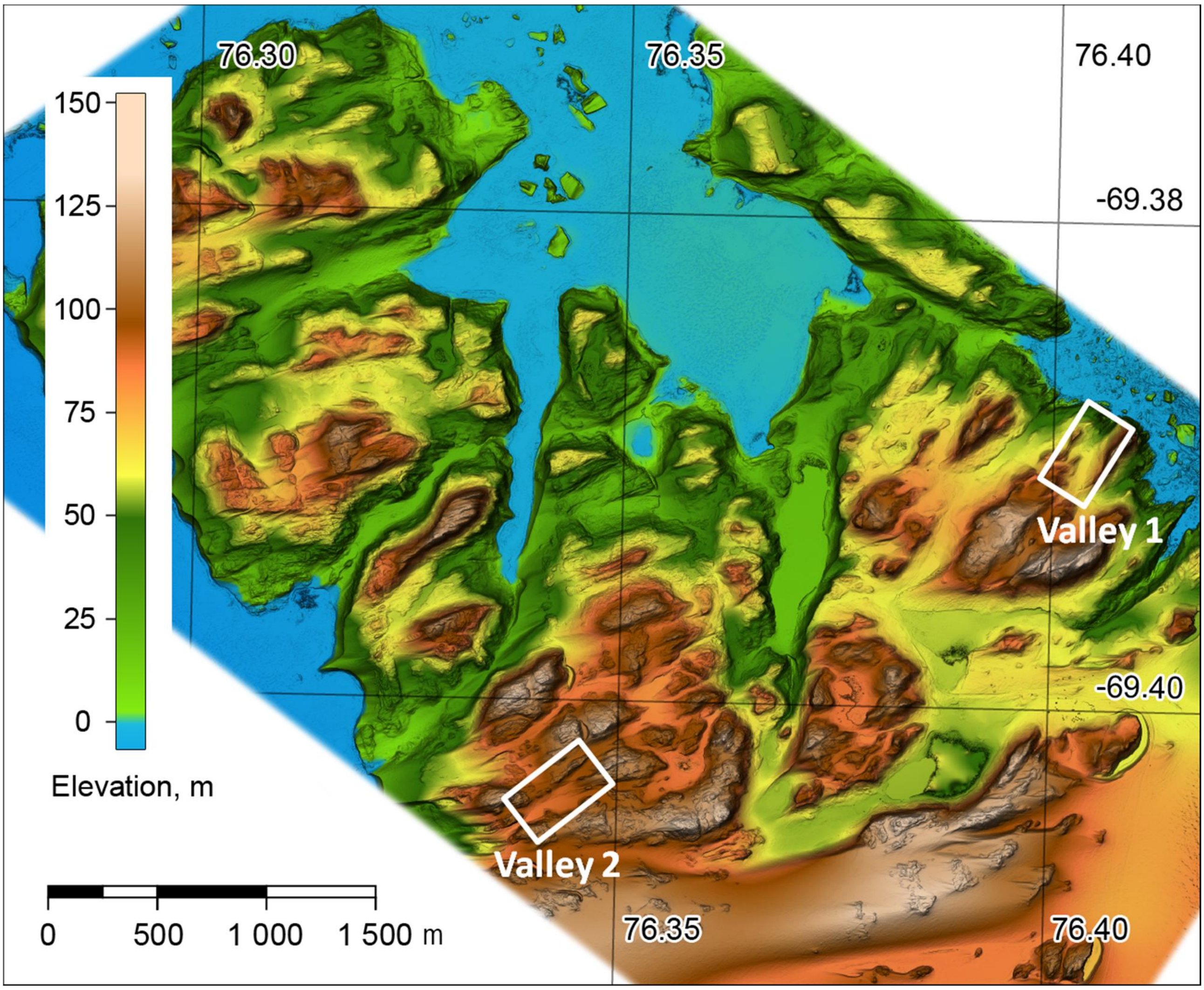

Figure S1 Location of the two key site valleys on the topographic map of the Brokness Peninsula in the Larsemann Hills, East Antarctica (the map was obtained from the mosaic of UAV images as of January 29, 2017).

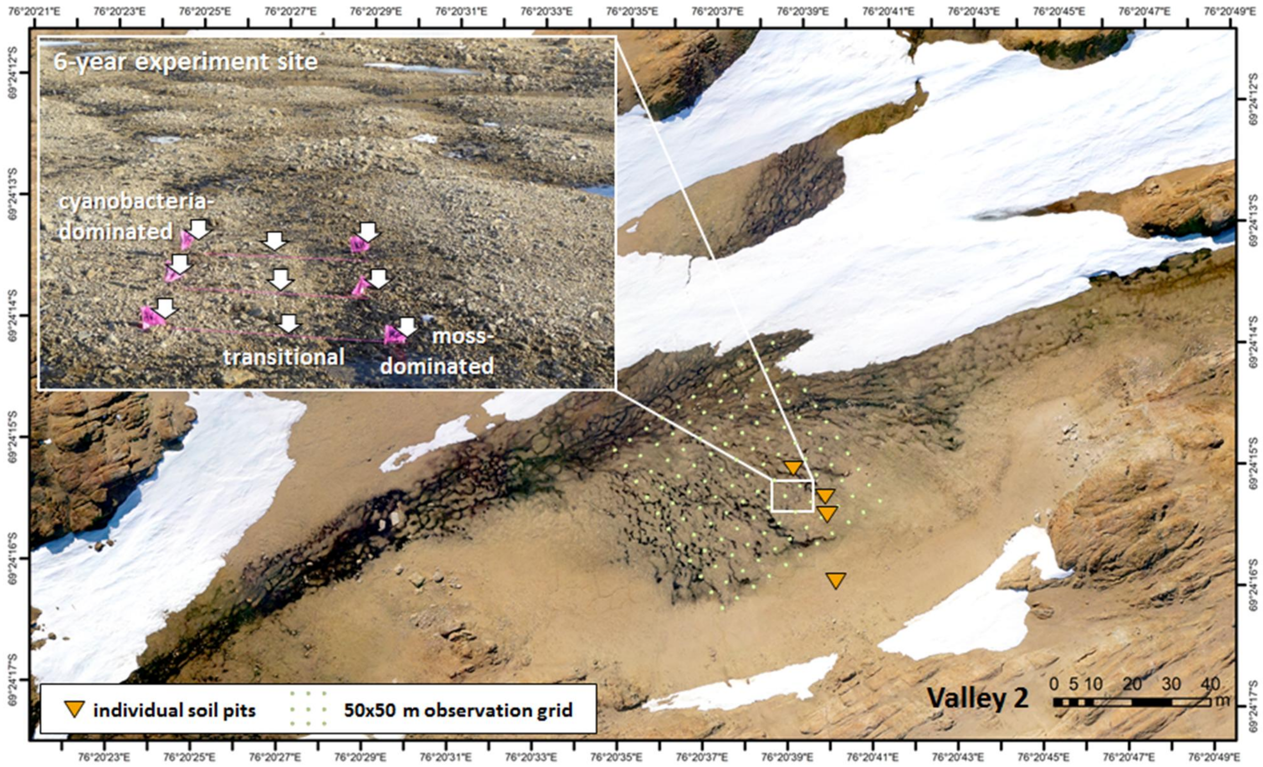

Figure S2 Location of the 6-year field experiment with the glass slides, individual soil pits and the 50x50 m BSC observation site in the Valley 2 (69.40422°S, 76.3438°E) at the Brokness Peninsula of the Larsemann Hills, East Antarctica.

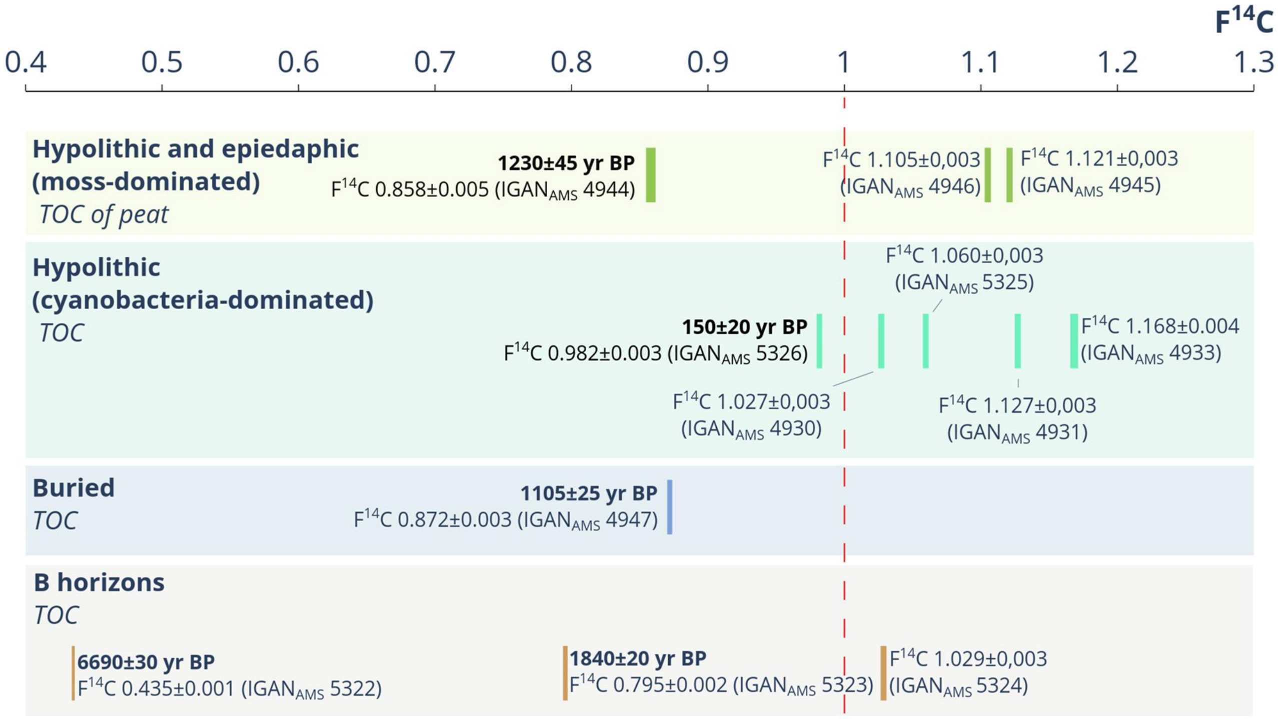

Figure S3 Fraction modern ($F^{14}C$, 1σ) and conventional ^{14}C age (1σ, yr BP) of the total organic carbon (TOC) in various topsoil and subsoil horizons in the valley floor biotopes of the Larsemann Hills. The values are sorted along the $F^{14}C$ data on the X-axis; the dashed line indicates $F^{14}C$=1 and the bars width corresponds to the 1σ ranges of $F^{14}C$.

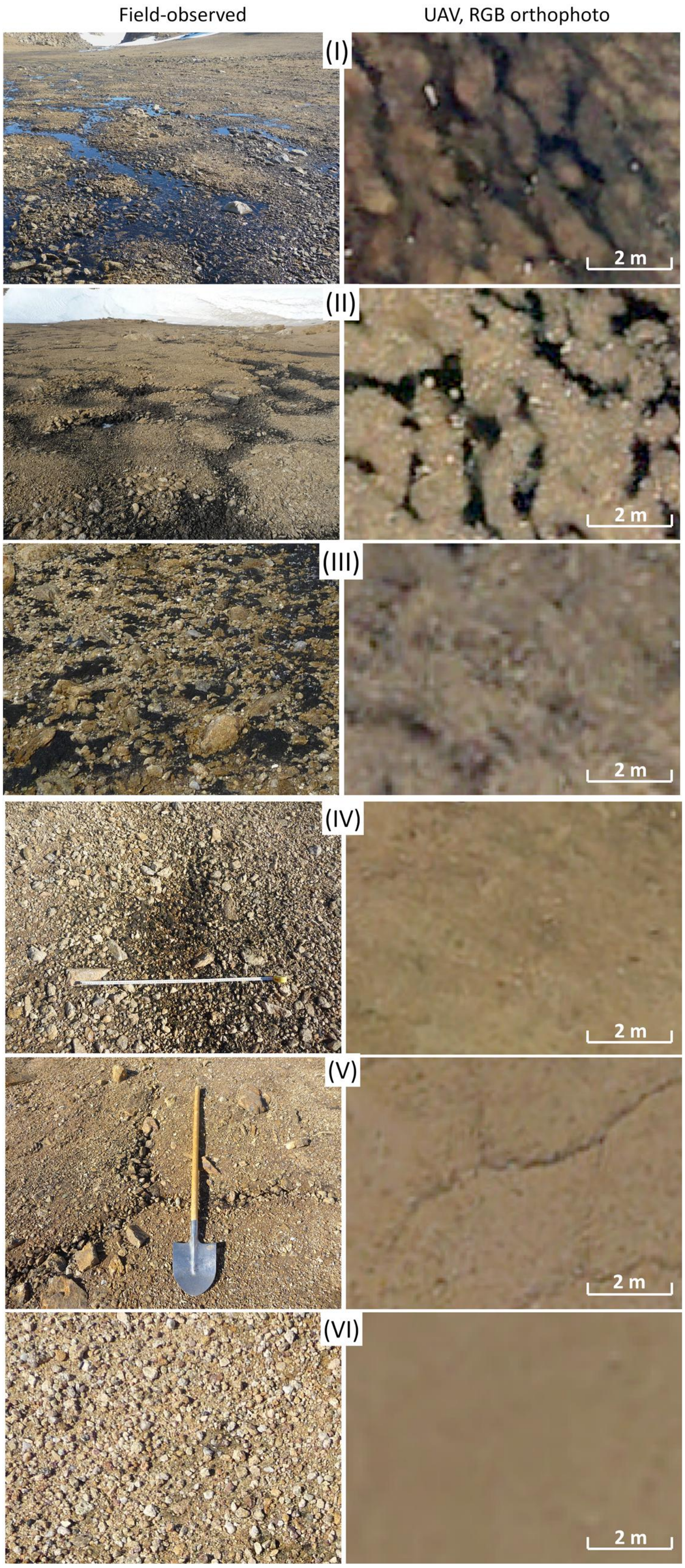

Figure S4 Examples of BSC classes and soil surfaces as observed in the field and their close analogues on the UAV orthophotos: (**I**) amphibian algae- and cyanobacteria-dominated (temporally wet locations); (**II**) amphibian algae- and cyanobacteria-dominated + epiedaphic moss-dominated (temporally dry locations); (**III**) hypolithic moss-dominated + epiedaphic moss-dominated; (**IV**) hypolithic cyanobacteria-dominated + hypolithic moss-dominated with patchy proliferation of epiedaphic moss; (**V**) hypolithic cyanobacteria-dominated; (**VI**) no BSC (barren ahumic soil) or ephemeral BSC .

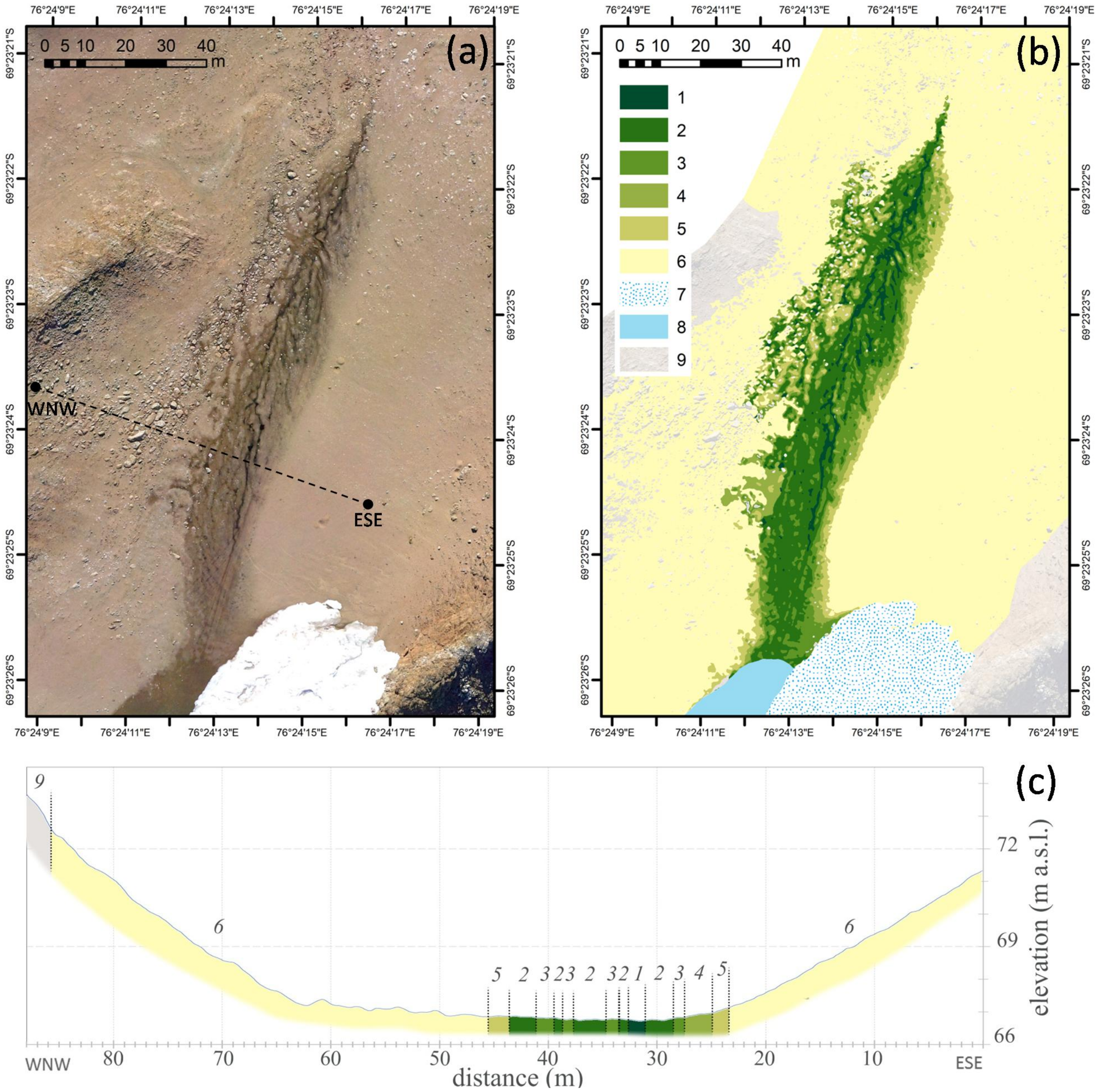

Figure S5 Distribution of BSC in the valley 1 (69.3901°S, 76.4039°E; Larsemann Hills): (**a**) orthophotomap obtained from mosaic of UAV images as of January 29, 2017; (**b**) distribution of BSC classes and other landscape components: 1 – amphibian algae- and cyanobacteria-dominated (class I); 2 – amphibian algae- and cyanobacteria-dominated + epiedaphic moss-dominated (class II); 3 – hypolithic moss-dominated + epiedaphic moss-dominated (class III); 4 – hypolithic cyanobacteria-dominated + hypolithic moss-dominated with patchy proliferation of epiedaphic moss (class IV); 5 – hypolithic cyanobacteria-dominated (class V); 6 – no BSC (barren ahumic soil) or ephemeral BSC (class VI); 7 – snow patches; 8 – meltwater; 9 – consolidated rock exposures and rock debris with epi/endolithic colonization; (**c**) DEM-derived geomorphological profile through the valley as indicated by dashed line on the orthophotomap, the colors and numerals match the legend on (**b**).

Table S1 TOC%, TN% and TOC/TN in the barren ahumic soils with no macroscopic traces of BSC.

ID	Soil	Location	GPS	Depth, cm	TOC, %	TN, %	TOC/TN
NSM 10-37	Barren ahumic soil	Valley 1	69°23'24.3"S 76°24'16.5"E	0-10	0.04	0.01	4
				10-20	0.05	0.01	5
				20-30	0.05	0.01	5
				30-40	0.04	0.01	4
				60-70	0.04	-	-
				70-80*	0.04	-	-
NSM 10-42	Barren ahumic soil	Valley 1	69°23'25.0"S 76°24'16.1"E	0-10	0.06	0.02	3
				10-20	0.07	0.01	7
				20-30	0.05	0.01	5
				30-40	0.06	0.01	6
				40-50	0.04	-	-
NSM 10-03-4	Barren ahumic soil	Valley 2	69°24'16.3"S 76°20'40.4"E	0-10	0.07	0.01	7
				10-20	0.05	0.01	5
				20-30	0.05	-	-
				30-40	0.04	-	-

*suprapermafrost horizon

Table S2 Statistics of properties for the selected spatially-adjacent hypolithic horizons.

Properties	Topography/ Morphotype	N	Mean	Median	Mini-mum	Maxi-mum	Std. Dev.	p-value Shapiro-Wilk α=0.05	p-value Mann Whitney U α=0.01
**TOC** %	Polygon	75	0.37	0.26	0.06	1.33	0.28	8.13E-08	2.22E-08
	Trough	54	1.07	0.63	0.16	4.69	1.03	8.35E-08	
**TN** %	Polygon	65	0.04	0.02	0.01	0.12	0.03	7.85E-07	4.18E-09
	Trough	46	0.11	0.06	0.02	0.38	0.09	2.27E-06	
**TOC** %	Moss-dominated	14	2.75	2.38	0.75	4.69	1.10	0.31193	
	Cyanobacteria-dominated	113	0.44	0.34	0.06	1.34	0.31	1.06E-08	
**TN** %	Moss-dominated	14	0.22	0.20	0.07	0.38	0.09	0.09926	
	Cyanobacteria-dominated	112	0.04	0.03	0.00	0.13	0.03	8.74E-09	
**TOC:TN**	Moss-dominated	14	12.74	12.65	10.80	14.70	1.18	0.63937	
	Cyanobacteria-dominated	98	15.25	12.10	4.00	43.40	7.53	1.55E-09	
$\delta^{13}C_{org}$ ‰	Moss-dominated	7	-27.14	-27.05	-27.87	-26.43	0.55	0.70411	
	Cyanobacteria-dominated	27	-26.64	-25.71	-30.04	-24.02	2.17	0.00238	
